# Evaluation of Linkage Disequilibrium Pattern and Association Study on Seed Oil Content in *Brassica napus* Using ddRAD Sequencing

**DOI:** 10.1371/journal.pone.0146383

**Published:** 2016-01-05

**Authors:** Zhikun Wu, Bo Wang, Xun Chen, Jiangsheng Wu, Graham J. King, Yingjie Xiao, Kede Liu

**Affiliations:** 1 National Key Laboratory of Crop Genetic Improvement, Huazhong Agricultural University, Wuhan, Hubei, China; 2 Southern Cross Plant Science, Southern Cross University, Lismore, NSW, Australia; USDA-ARS-SRRC, UNITED STATES

## Abstract

High-density genetic markers are the prerequisite for understanding linkage disequilibrium (LD) and genome-wide association studies (GWASs) of complex traits in crops. To evaluate the LD pattern in oilseed rape, we sequenced a previous association panel containing 189 *B*. *napus* inbred lines using double-digested restriction-site associated DNA (ddRAD) and genotyped 19,327 RAD tags. A total of 15,921 RAD tags were assigned to a published genetic linkage map and the majority (71.1%) of these tags was uniquely mapped to the draft reference genome “Darmor-*bzh*.” The distance of LD decay was 1,214 kb across the genome at the background level (*r*^2^ = 0.26), with the distances of LD decay being 405 kb and 2,111 kb in the A and C subgenomes, respectively. A total of 361 haplotype blocks with length > 100 kb were identified in the entire genome. The association panel could be classified into two groups, P1 and P2, which are essentially consistent with the geographical origins of varieties. A large number of group-specific haplotypes were identified, reflecting that varieties in the P1 and P2 groups experienced distinct selection in breeding programs to adapt their different growth habitats. GWAS repeatedly detected two loci significantly associated with oil content of seeds based on the developed SNPs, suggesting that the high-density SNPs were useful for understanding the genetic determinants of complex traits in GWAS.

## Introduction

Genome wide association studies (GWASs) utilize historical recombination events and linkage disequilibrium (LD) in diverse germplasm to detect sequence variants associated with phenotypic traits. The approach has proven to be a powerful tool for exploring the genetic architecture of agronomically important traits in crop species [1–7]. A prerequisite of GWAS is the comprehensive characterization of the LD pattern across the entire genome of the germplasm resources. The extent of LD not only determines the number of markers needed to saturate the genome, but also influences the resolution of GWAS [8]. Genome-wide LD patterns reflect the history of recombination and selection during evolution and artificial breeding of existing germplasm resources [9], and so may introduce spurious associations in GWAS [10].

The extent and pattern of LD in *B*. *napus* have primarily been interrogated using several hundred conventional molecular markers including simple sequence repeat (SSR) and amplified fragment length polymorphism (AFLP) markers. The mean *r*^*2*^ value was estimated to be 0.023 for linked loci and 0.019 for unlinked loci in a collection of 509 *B*. *napus* inbred lines with 89 SSR markers [11]. A similar LD level was estimated in a set of 172 accessions comprised of 69 cultivars and 103 synthetic lines with 96 SSR markers [12]. The distance of LD decay was estimated to be within 2 cM amongst 85 canola quality winter rapeseed genotypes using 845 AFLPs [13], or to be 0.5–1 cM in 192 diverse inbred lines using 451 SSR markers [14]. However, the limited number of markers used in these studies may give inaccurate estimates of LD level and LD decay in allotetraploid *B*. *napus*. Thus, the extent and LD decay in *B*. *napus* were re-evaluated recently using high-density SNP arrays. The distance of LD decay was about 0.6 cM (*r*^*2*^ < 0.2) for the A and C subgenomes in 313 inbred lines as revealed by Infinium array containing 4,329 SNPs [15]. With the *Brassica* 60 K SNP array, the distances of LD decay in the A and C subgenomes were estimated to be 210 and 810 kb (*r*^*2*^ < 0.2) in a collection of 472 rapeseed accessions, respectively [16], while they were 250–300 kb and 2000–2500 kb (*r*^*2*^ < 0.1) in 203 Chinese semi-winter rapeseed breeding lines, respectively [17]. Although the *Brassica* 60 K Infinium SNP array is a good tool for genotyping, it remains expensive for a regular laboratory when the large population needs to be genotyped.

With the advent of next generation sequencing (NGS) technologies and significant reduction of sequencing cost, SNPs are being discovered and genotyped in high-throughput manners such as whole genome resequencing (WGR) and double-digested restriction-site associated DNA (ddRAD) using the Illumina NGS platform. The NGS technology coupled with reduced representation libraries (RRLs) is an economic way to efficiently discover SNPs in large scale [18]. In diploid species, SNP calling is commonly performed by aligning read-sequence to reference genome [19]. However, SNP discovery using NGS remains a challenge in polyploidy species because these species such as oilseed rape usually have complex genomic structure as well as numerous paralogs, homeologs and repetitive sequences [20, 21], or without a reference genome sequence. To solve these problems, Lu et al. [22] proposed a reference-independent Universal Network Enabled Analysis Kit (UNEAK) pipeline to discover SNPs from genotyping by sequencing (GBS) data. The UNEAK pipeline only made use of two reciprocal tags within a network with the aim to avoid paralogue’s confounding, but it is still a challenge to effectively ruling out false identification of SNP loci in natural germplasm with complex genomes. Thus, an efficient method that could distinguish allelic SNPs from hemi-SNPs between homologous sequences will be urgently important for reducing the false positive rate in SNP calling as well as significantly increasing the numbers of informative SNPs.

In this study, we sequenced an association panel with 189 *B*. *napus* inbred lines using ddRAD. A total of 19,327 polymorphic tags containing 31,833 SNPs were discovered and genotyped using the UNEAK pipeline with modifications. Of which, 15,921 tags were assigned to a published genetic linkage map through LD-based mapping. The genetic linkage showed a good collinearity to the reference genome of the *B*. *napus* cultivar ‘Darmor-*bzh*’ [20]. Using this set of SNP markers, we comprehensively characterized the population structure and LD patterns across the entire genome of *B*. *napus*, and test their usefulness for GWAS with seed oil content.

## Materials and Methods

### Plant materials and trait measurement

A previously described association panel consisting of 189 *B*. *napus* inbred lines was used for this study (S1 Table) [14]. They were planted in the experimental field at Huazhong Agricultural University, Wuhan, China, in the winters of 2008, 2009 and 2010, and harvested in the springs of 2009, 2010 and 2011. Field trials followed a completely randomized block design with three replicates each year. Field management followed the standard agricultural practice. At mature stage, the seeds of normally developed plants from middle were harvested and 3–5 individuals were used to measure seed oil content using near infra-red spectroscopy (NIRS) [23].

### Library construction and ddRAD sequencing

Genomic DNA was extracted from young leaves of a single plant for each inbred line. ddRAD libraries for all inbred lines were constructed as previously described [21]. Briefly, 200 ng genomic DNA was digested with *Sac*I and *Mse*I. Restriction fragments for each individual were then ligated to the SacAD and MseAD adaptors with unique barcodes. The final ligates of 12 individuals were pooled to form a library and separated on a 2% agarose gel. Fragments in a size range between 220 and 500 bp, which corresponded to restriction fragments in a size range of 140–420 bp before adaptor ligation, were recovered from the gel and purified with a Qiagen gel purification kit (Qiagen Inc., Valenica, CA, USA). The pooled libraries were amplified and PCR products were separated on a 2% agarose gel. Fragments in a size range of 270–550 bp were purified with a Qiagen gel purification kit and submitted for sequencing on a HiSeq2000 platform with paired-end (PE) reads of 90 bp.

### SNP discovery and genotyping

**Fig 1:**
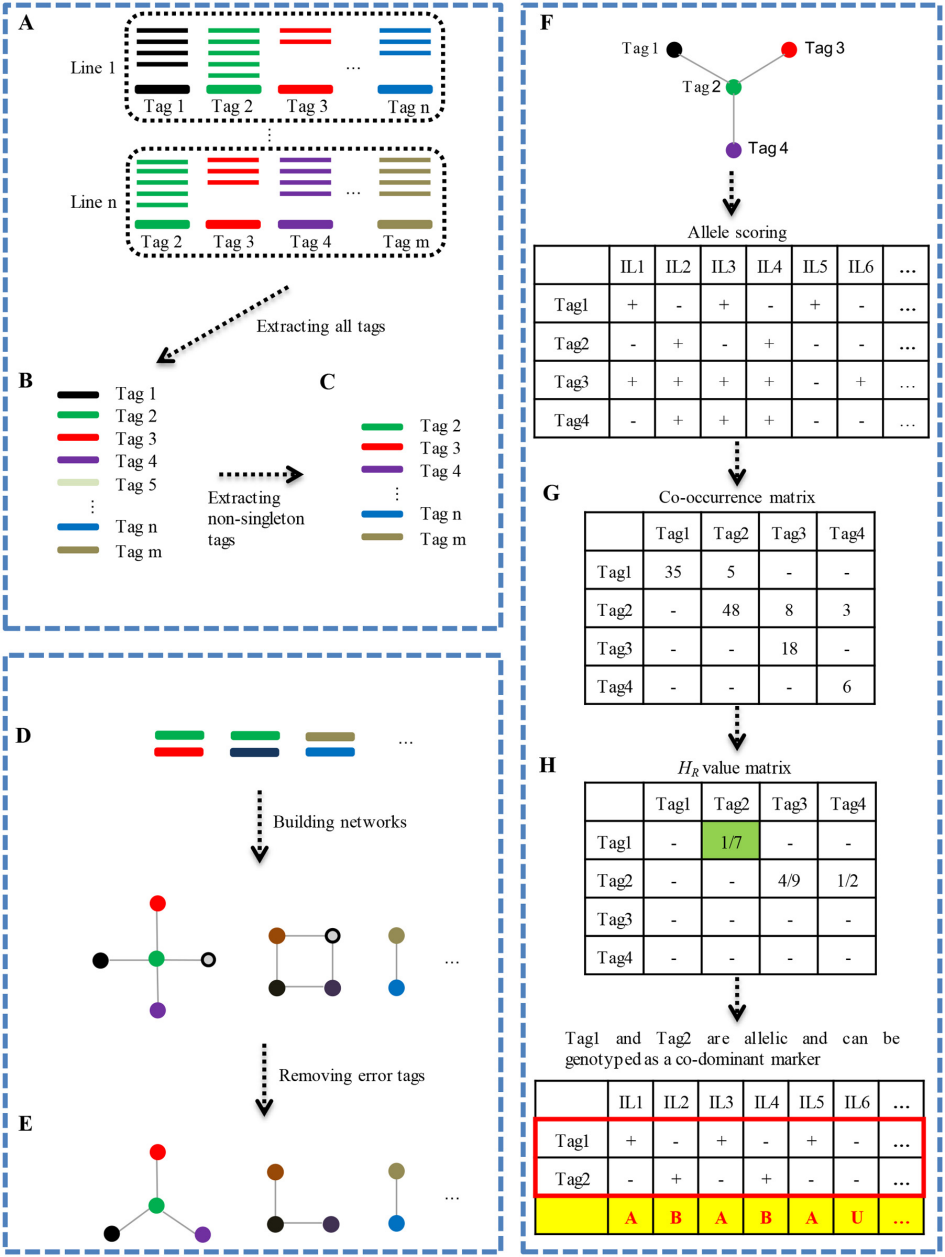
Workflow of the modified UNEAK pipeline. **(A)** Short reads are collapsed to form tags for each inbred line. **(B)** The tags from all inbred lines in the association panel are collapsed again. **(C)** The tags only from a single inbred line are removed as sequencing errors. Only tags from more than one inbred line are kept for further analysis. **(D)** Pairwise alignment is performed between any two tags to form networks with at most two mismatches on each end of PE reads. **(E)** By employing the parameter error tolerance rate (ETR), the error tags (shaded circles) are removed from networks. **(F)** Scoring of allelic tag pairs using a network as an example. Each tag in the network is scored as an allele. “+” and “-” represent the presence and absence of the corresponding allele in an inbred line and then tabulate for the association panel. **(G)** The co-occurrence or combination of any two alleles from a locus within a network formed a possible genotype. And a co-occurrence matrix containing all possible homozygous and heterozygous genotypes is created. **(H)** Allelic tag pairs are discriminated from complicated networks mixed with homologous tags using relative heterozygosity (*H*_*R*_). The tag pairs with *H*_*R*_ smaller than the empirical value (0.2) are considered as the allelic tags and then genotyped for each inbred line.

The 90 bp PE reads that passed quality filtering were parsed into different individuals, with the outermost barcodes and remnant restriction sites at both ends exactly matching the adaptors used. The clean sequence data was submitted to National Center for Biotechnology Information (NCBI) with the project SRP066134. After trimming the 5 bp barcode and the 5 bp on the 3’ end of sequences, the remaining 80 nucleotides of each PE read were kept for further analysis. A modified UNEAK pipeline included an additional step to discriminate allelic tags (Fig 1). Briefly, identical PE reads of each inbred line were firstly collapsed into tags (Fig 1A). The tags from all inbred lines in the association panel were then pooled and collapsed again to form clusters and keep the ones that contained unique tags from more than one inbred line (Fig 1B and 1C). The unique tags that contained only a single PE read were removed as sequencing errors. The remaining unique tags were aligned each other to build networks. Two mismatches were allowed on each end of the PE reads (Fig 1D). Error tags were further filtered from the networks using the parameter of error tolerance rate (ETR), as done by Lu et al. [22] (Fig 1E). The networks harboring two to ten tags were kept for allelic tags discrimination.

Allelic tags in a network were discriminated from paralogous and/or homologous tags with an additional module. Each unique tag in a network was treated as an allele, and designated as A1, A2, A3, etc. Tags were scored as “+” or “-” according to the presence or absence of the corresponding allele in each inbred line, and tabulated in the association panel (Fig 1F). The co-occurrence or combination of any two alleles from a locus within a network formed a possible genotype. A co-occurrence matrix was then created to indicate the number of all possible homozygous and heterozygous genotypes (Fig 1G). The co-occurrence matrix was then converted into a relative heterozygosity (*H*_*R*_) value (Fig 1H), which is defined as the ratio of heterozygotes to the smaller number of homozygotes. The *H*_*R*_ value is used to distinguish allelic tags from homologous tags. In theory, the *H*_*R*_ value should be very low for each network when excluding the paralogues and homologues in a population of inbred lines. We assumed that the observed tag pairs with low *H*_*R*_ values are putative allelic tag pairs. The *H*_*R*_ is empirically set to 0.20 to eliminate paralogous or homologous tag pairs, based on the *H*_*R*_ distribution of all available tag pairs. If the *H*_*R*_ of a specific tag pair was smaller than the threshold, the two tags were considered to be allelic. Otherwise, they were considered to be from homologous loci.

### LD-based genetic mapping

Multiple SNPs from a tag should be in a complete LD due to the small insert size (< 420 bp). Thus one SNP was selected from each allelic tag pair to represent the locus to reduce computational load. All discovered SNPs were directly aligned to the BnaNZDH genetic linkage map constructed using SNPs developed with the same ddRAD protocol [21]. The SNPs on the genetic linkage map were used as anchor SNPs for mapping the unassigned SNPs via a LD-based genetic mapping method [24, 25]. If the LD (*r*^*2*^) between an anchor SNP and an unassigned SNP is larger than the background LD, these two SNPs are thought to be linked. The background LD level was set to the 99^th^ percentile of *r*^2^ distribution for unlinked anchor markers [14].

### Population stratification and genetic diversity

To reduce the influence of strong LD on the assessment of population stratification, a set of SNPs were selected by setting an LD (*r*^2^) threshold of 0.4 for SNP pairs in a sliding window of 50 SNPs using PLINK [26]. A Bayesian model-based structure analysis [27] and principal component analysis [28] were done using the selected SNPs to infer the population structure in the 189 diverse *B*. *napus* lines. PIC values were calculated for each SNP using PowerMarker v3.25 [29]. The differences of PIC between groups and subgenomes were evaluated using two sampled *t* test in R [30].

### LD and haplotype block analyses

LD was estimated as the correlation coefficient *r*^2^ [31] between pairs of mapped SNPs having minor allele frequency (MAF) ≥ 0.05 using a custom Perl script. The plot depicting LD decay with physical distances was drawn using non-linear regression in R according to Gaut and Long [31]. LD decay distance was determined when *r*^2^ fell to the background LD (*r*^2^ = 0.26).

The estimation of haplotype blocks was performed using PLINK [26] with a window size of 10 Mb. The haplotype blocks with high LD were identified according to the confidence interval (CI) model [32]. Haplotype blocks shorter than 100 kb were removed. Haplotype frequency was calculated using a custom Perl script. If a haplotype block is only observed in one group but not in the other group, it is considered to be group-specific block. If a haplotype block is simultaneously detected in both groups, the haplotype block is considered to be common block. If the common haplotype block has a large frequency difference (>0.40), it is considered to be group-preferential block.

### Association study of seed oil content in *B*. *napus*

Broad-sense heritability of seed oil content was calculated as $H^{2}=\delta_{\text{g}}^{2}/\left(\delta_{\text{g}}^{2}+\delta_{\text{ge}}^{2}/\text{n}+\delta_{\text{e}}^{2}/\text{nr}\right)$, where $\delta_{\text{g}}^{2}$ is the genetic variance, $\delta_{\text{ge}}^{2}$ is the variance due to G × E interaction, $\delta_{\text{e}}^{2}$ is the residual error, n is the number of environments and r is the number of replicates within environment. The estimates of $\delta_{\text{g}}^{2}$, $\delta_{\text{ge}}^{2}$, $\delta_{\text{e}}^{2}$ were obtained from the analysis of variance (ANOVA) using the SAS v9.2 [33]. Within each year, the average seed oil content for each inbred line represented the phenotypic value. The best linear unbiased prediction (BLUP) of each line, which was estimated using the MIXED procedure in SAS v9.2 by treating genetic effect as random effect [34], represented the phenotypic value of three years. The distribution of oil content was tested for normality using Shapiro-Wilk test in R. The phenotypic values from averages within each year and BLUPs from three years were used for genome-wide association mapping.

GWAS was performed with the mixed linear model (MLM) with the control of pairwise kinship among 189 inbred lines using Genome-wide Efficient Mixed Model Association (GEMMA) [35]. The genome-wide significance threshold of GWAS was set as 9.7×10^-5^ (1/n, n is number of markers) based on the adjusted Bonferroni correction method [36]. The quantitative trait locus (QTL) identified by GWAS was designated with initial letters “*qOC*” followed by alphabetical letter of the chromosome, on which the QTL located. For the repeatedly detected QTLs, based on all significant SNPs sorted by physical positions in each locus, the haplotype, which at least had two inbred lines, and corresponding number of inbred lines and average oil content were investigated. Stepwise regression was performed to examine the effect of multiple markers of all QTLs in each year and estimate total variance explained (*R*^2^) [4]. The forward–backward (stepwise) selection of markers were on the basis of *F* test for each marker with *P*-value < 0.05.

## Results

### Sequencing and SNP calling in diverse *B*. *napus* inbred lines

The ddRAD libraries for the 189 *B*. *napus* inbred lines were constructed as previously described [21] and then sequenced with PE reads of 90 bp. A total of 506.81 million 90 bp PE reads containing barcodes and partial recognition sequences were generated. The number of reads per inbred line ranged from 1.13 to 6.33 million, and the average number of reads per inbred line was 2.68 million. For each read, the first 5 bp index at the 5’ end and the last 5 bp error-enriched nucleotides at the 3’ end were trimmed. Therefore, 80 bp PE reads were kept for the following analysis.

Firstly, identical 80-bp PE reads are collapsed into tags (Fig 1A–1C). These tags are clustered into networks by pairwise alignment with at most two mismatches on each end of the 80-bp PE read (Fig 1D). After removing error tags and filtering networks only having a single tag, a collection of 90,235 networks with two tags, 52,153 networks with three to 10 tags, and 7,484 networks with more than 10 tags were obtained (S2 Table). With the assumption that all loci within inbred lines are almost homozygous with very low *H*_*R*_, we discriminated allelic tag pairs from paralogous or homologous tags in a network as previously described [21]. Finally, 19,327 allelic tag pairs containing 31,833 SNPs were identified and genotyped in the association panel, which included 10,918 (56.5%) allelic tag pairs from two-tag networks and 8,409 (43.5%) allelic tag pairs from complex networks with multiple tags. One SNP was selected from each allelic tag pair to represent the locus, which resulted in a set of 19,327 SNPs for the following analysis.

### Assignment of SNPs to a genetic linkage map

To determine chromosomal positions of the 19,327 SNPs, we aligned them to a genetic linkage map constructed using the same ddRAD protocol in the BnaNZDH population [21]. A subset of 4,995 SNPs each had a corresponding SNP marker on the genetic linkage map, while the remaining 14,332 SNPs did not have a corresponding marker in the genetic linkage map. To assign the 14,332 SNPs to the genetic linkage map, we employed the LD-based genetic mapping strategy [24,25] by using the 4,995 mapped SNP markers as anchors. The whole-genome LD values (*r*^2^) of unlinked marker pairs of the 4,995 anchor SNPs were calculated, and the 99^th^ percentile of the *r*^2^ distribution in the association panel was 0.26, which was set as the background LD (S1 Fig). In general, LD is an indicator of the tightness of linked markers. The larger the LD, the closer the genetic or physical distance between marker pairs [8]. A pair of markers with LD value larger than the background LD was considered to be linked. Pairwise LD values (*r*^*2*^) were calculated between the 14,332 unmapped SNP and the 4,995 anchor SNPs, and the anchor marker having the highest LD value was selected to represent the position of the unmapped SNP. Using LD-based genetic mapping, we further assigned 10,926 SNPs to the BnaNZDH linkage map. In total, 15,921 SNPs were assigned to the genetic linkage map, with 6,883 (43.2%) and 9,038 (56.8%) SNPs located in the A and C subgenomes, respectively (S3 Table). These SNPs were unevenly distributed on chromosomes, especially on chromosomes in the C subgenome. The average number of SNPs per chromosome in the A subgenome is 688, varied from 472 on chromosome A2 to 1,053 on A3. The average number of SNPs per chromosome in the C subgenome is 1,004, varied from 332 on C5 to 1,850 on C3 (S3 Table).

**Fig 2:**
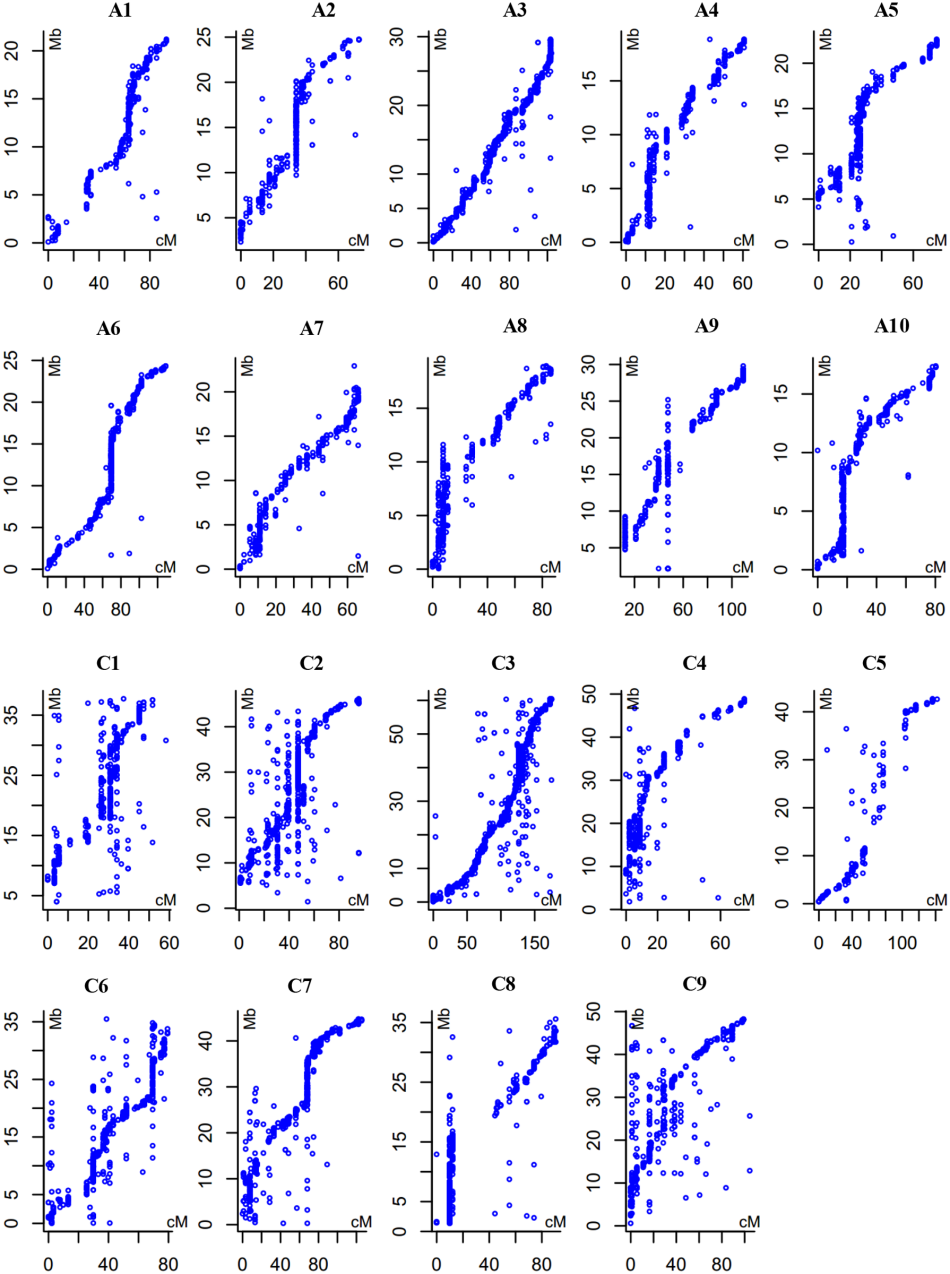
Alignments between the LD-based linkage map and the *B*. *napus* reference genome. The horizontal axis represents the genetic position (cM) of the SNPs on the genetic linkage map based on the BnaNZDH population [21] and LD-based mapping. The vertical axis represents the physical position (Mb) according to the *B*. *napus* reference genome ‘Darmor-*bzh*’ [20].

The physical positions of the 15,921 SNPs were determined by homology search against the draft reference sequence of the *B*. *napus* cultivar ‘Darmor-*bzh*’ [20] with an E value ≤ 1×10^-15^. Out of which, 11,326 SNPs were aligned to a unique position on the reference genome, while the remaining 4,595 SNPs either aligned to multiple positions on the reference genome or did not have a hit with an E value ≤ 1×10^-15^. Moreover, the genetic positions of the aligned SNPs showed good collinearity with the *B*. *napus* reference genome (Fig 2), indicating that LD-based genetic mapping is a feasible method for the determination of the putative genetic or physical positions of molecular markers.

### LD patterns and haplotype blocks in the *B*. *napus* genome

From the assigned 11,326 SNPs, the 10,343 SNPs with a MAF higher than 0.05 were selected for LD pattern analysis. Genome-wide LD was calculated using the squared allele correlation (*r*^2^) between pairs of the 10,343 SNPs [31]. The genome-wide average LD (*r*^2^) was 0.113. The average LD in the A subgenome was 0.075, which is significantly lower than that in the C subgenome (0.132) (*P* < 0.001). LD levels showed considerable variations between chromosomes. In the A subgenome, the LD varied from 0.028 (A3) to 0.154 (A6), while it varied from 0.039 (C3) to 0.217 (C8) in the C subgenome (Table 1). In the A subgenome, 7.41% intra-chromosomal SNP pairs showed LD (*r*^2^ > 0.26), significantly lower than the percentage in the C subgenome (14.64%, *P* < 0.001) (S4 Table).

**Table 1:** LD (*r*^*2*^) and LD decay distance along chromosomes.

Chr.	LD^a^	LD decay (kb)^b^	Chr.	LD	LD decay (kb)
A1	0.045	171	C1	0.176	2,587
A2	0.072	328	C2	0.201	4,089
A3	0.028	106	C3	0.039	329
A4	0.050	111	C4	0.200	3,867
A5	0.070	188	C5	0.063	227
A6	0.154	1,908	C6	0.074	791
A7	0.040	106	C7	0.075	933
A8	0.089	364	C8	0.217	3,341
A9	0.099	598	C9	0.081	692
A10	0.059	106			
A subgenome	0.075	405	C subgenome	0.132	2,111
Genome	0.113	1,214			

^a^ Average *r*^2^ values of all intra-chromosomal pairwise SNPs aligned to the reference genome sequence of the *B*. *napus* cultivar ‘Darmor-*bzh*’.

^b^ Physical distances of LD decay to *r*^2^ < 0.26.

**Fig 3:**
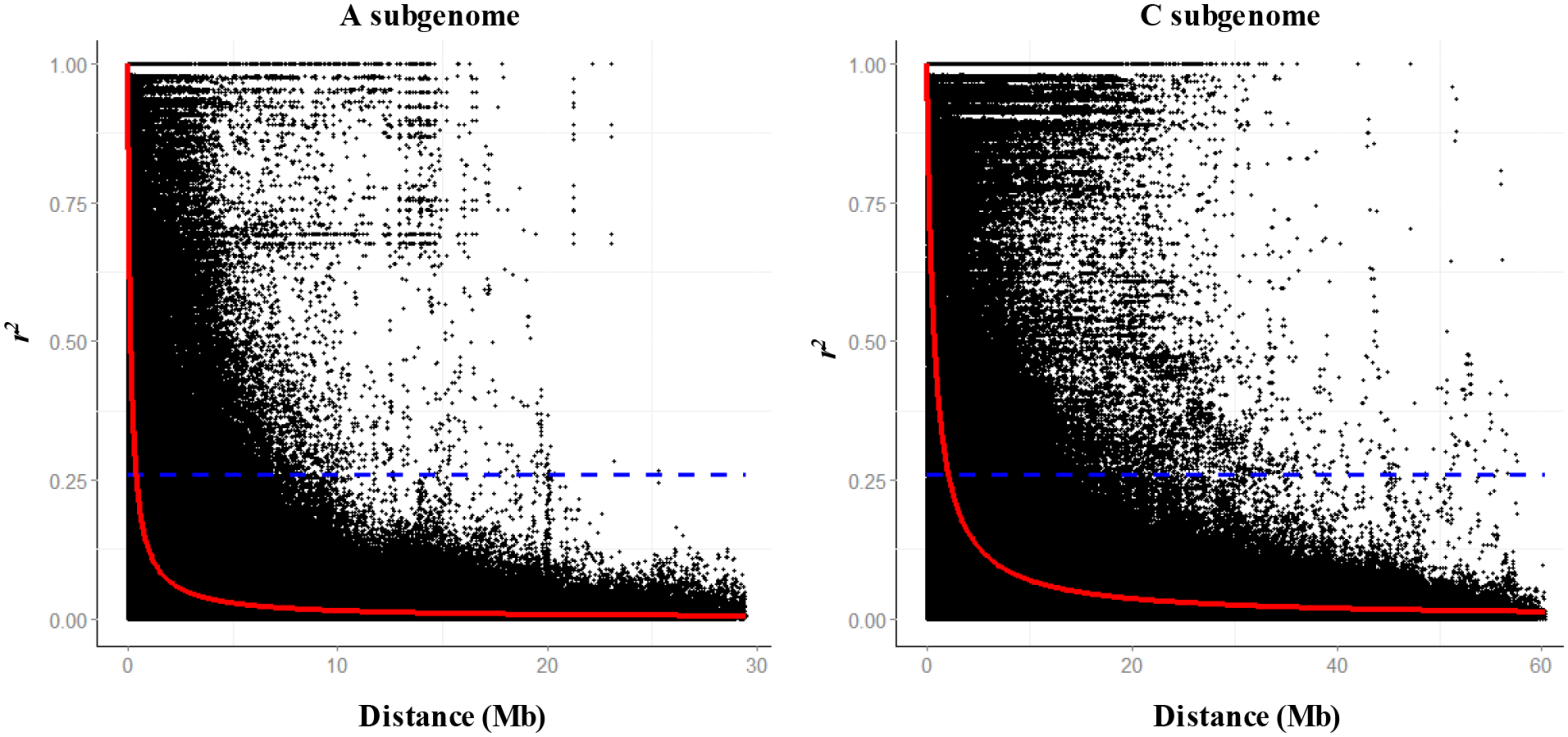
LD decay in the A and C subgenomes in the 189 *B*. *napus* diverse lines. The black dots indicate the *r*^*2*^ values of all SNP pairs within chromosomes for each subgenome. The red curve represents a nonlinear function of *r*^2^ against the SNP physical distance (Mb). The horizontal blue dash line indicates the estimated background level of LD (*r*^2^ = 0.26).

**Fig 4:**
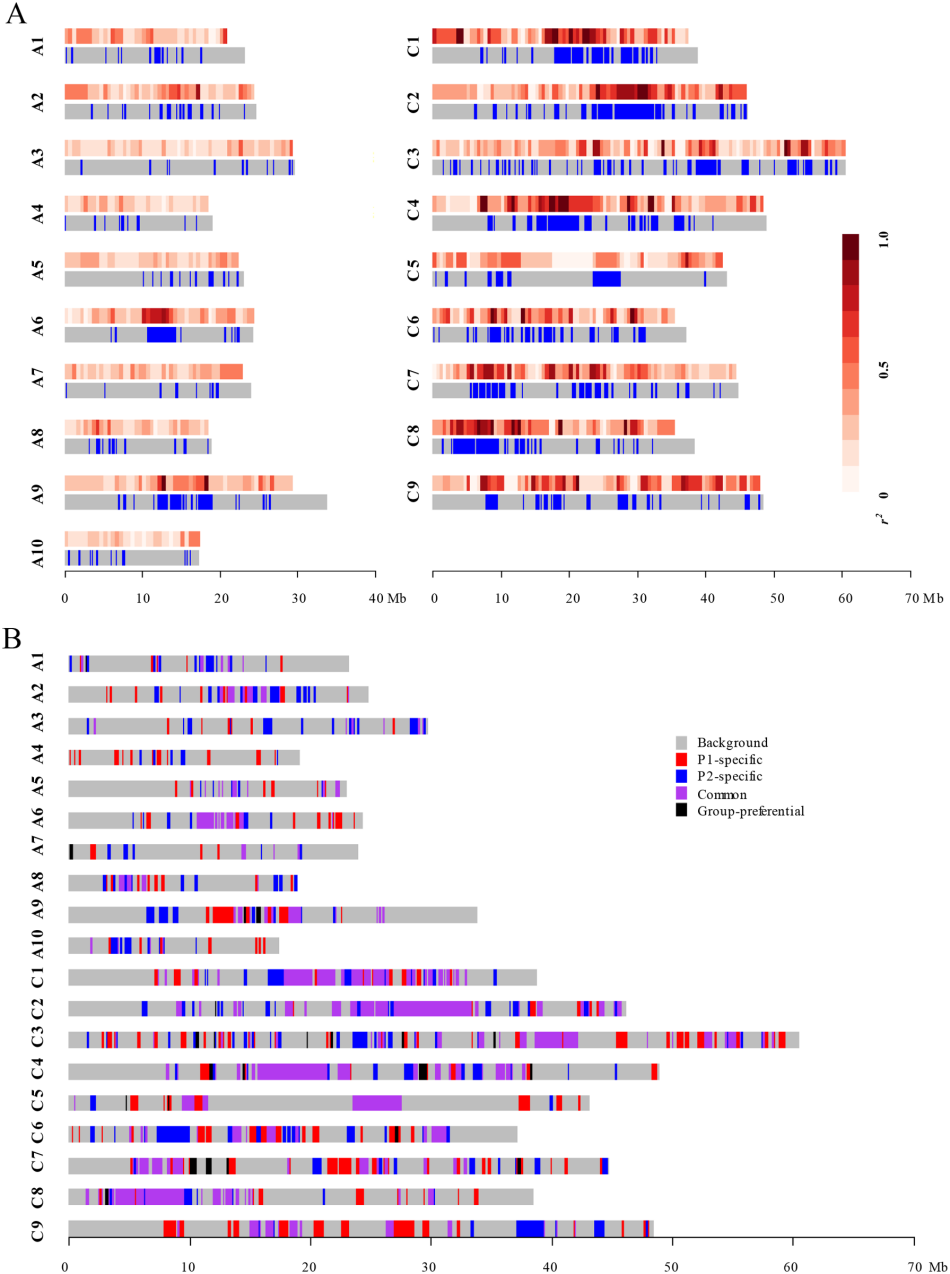
Genome-wide distribution of haplotype blocks for the entire panel and two groups in the association mapping panel. **(A)** The heatmap above indicates LD distribution. The color density represents the average *r*^2^ values of all SNP pairs in a sliding-window of 500 kb. The map below indicates the distribution of haplotype blocks with length > 100 kb. The gray color represents the background and the blue color indicates the haplotype blocks. **(B)** The distributions of different types of haplotype blocks for the P1 and P2 groups across each chromosome. Gray rectangles represent genomic regions that don’t contain any haplotype block. Red rectangles represent the P1-specific haplotype blocks and blue rectangles represent the P2-specific haplotype blocks. Orchid rectangles represent the common haplotype blocks with frequency difference ≤0.4, and black rectangles represent the group-preferential haplotype blocks with frequency difference >0.4.

The whole-genome LD decayed to 1,214 kb when *r*^2^ decreased to the background LD (*r*^2^ > 0.26). Significant variations in LD decay distances were observed among chromosomes. In the A subgenome, the distances of LD decay varied among chromosomes ranging from 106 kb (A3, A7 and A10) to 1,908 kb (A6), with an average LD decay distance of 405 kb. In the C subgenome, the distance of LD decay varied from 227 kb (C5) to 4,089 kb (C2), with an average LD decay distance of 2,111 kb (Table 1, Fig 3). And we performed a genome-wide haplotype block analysis to profile the pattern of LD variation across the whole genome. A total of 361 haplotype blocks extended for more than 100 kb along chromosomes were identified in the whole genome (Table 2, Fig 4A), with 118 and 243 haplotype blocks distributed in the A and C subgenomes, respectively. These haplotype blocks covered a total length of 145.1 Mb out of the 645.4 Mb anchored *B*. *napus* reference sequence [20]. The 118 haplotype blocks covered a total length of 32.8 Mb of the A subgenome, while the 243 haplotype blocks covered a total length of 112.3 Mb of the C subgenome.

**Table 2:** Length of haplotype blocks (HBs) along chromosomes in *B*. *napus* genome.

Chr.	Entire panel		Group-specific and—preferential HBs (kb)		
	No.^a^	Sum (kb)^b^	P1-specific^c^	P2-specific	Common (Preferential)^d^
A1	14	2,601	631	2,198	1,071(70)
A2	15	4,047	1,904	3,260	1,941(0)
A3	11	1,868	869	3,184	1,063(0)
A4	9	2,002	2,590	971	0
A5	11	2,838	1,003	531	1,383(0)
A6	9	5,499	2,152	1,185	3,006(0)
A7	8	2,052	955	1,197	987(227)
A8	10	2,626	1,636	2,071	1,443(0)
A9	18	7,422	3,321	2,623	3,110(485)
A10	13	1,893	1,284	1,656	486(0)
C1	25	12,365	2,620	3,186	10,255(0)
C2	32	16,707	1,512	4,516	13,887(127)
C3	49	17,588	8,366	4,724	9,593(1,009)
C4	24	14,187	2,346	3,343	12,447(1,209)
C5	11	7,380	3,505	786	5,873(235)
C6	27	10,713	4,991	6,336	4,470(296)
C7	27	11,977	5,442	3,022	6,970(1,390)
C8	25	11,475	2,357	1,422	9,247(238)
C9	23	9,903	7,389	3,989	4,007(0)
A subgenome	118	32,849	16,347	18,875	14,488(782)
C subgenome	243	112,297	38,528	31,322	76,749(4,504)
Genome	361	145,146	54,875	50,197	91,237(5,286)

^a^ The number of haplotype blocks longer than 100 kb on each chromosome.

^b^ The total length of haplotype blocks longer than 100 kb for each chromosome.

^c^ The total length of P1-specific haplotype blocks with each length longer than 50 kb.

^d^ The total length of common haplotype blocks that were simultaneously observed in two groups (outside parenthesis) and the total length of group-preferential haplotype blocks that simultaneously existed in two groups but with frequency difference > 0.4 (inside parenthesis).

**Fig 5:**
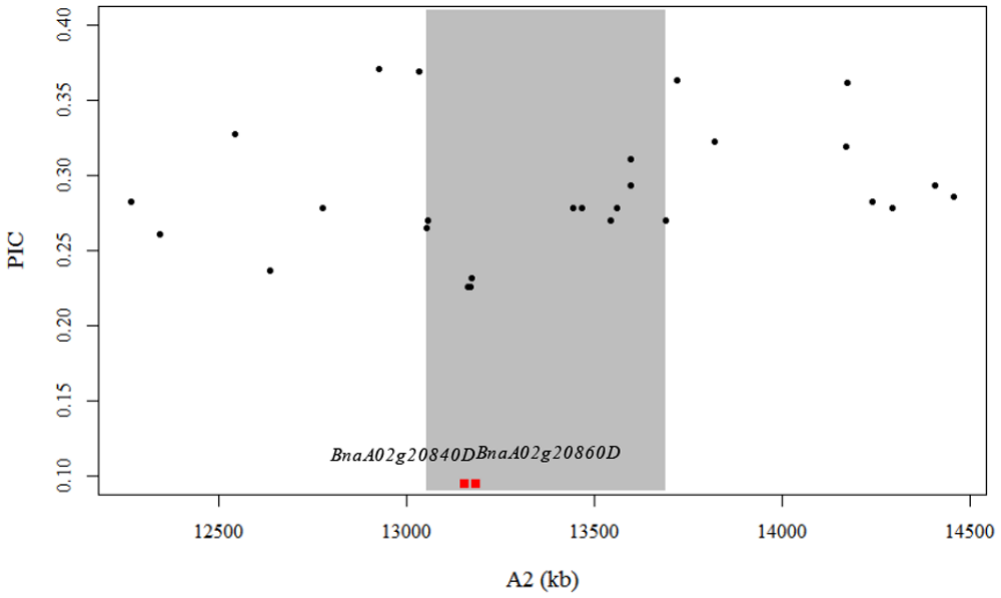
The haplotype block associated with genes controlling glucosinolate content. The gray windows represent the haplotype block for the entire panel, and the black dots indicate the PIC values of the SNPs in these region. The red boxes and the corresponding text indicate the positions and names of the genes possibly associated with the forming of the haplotype block.

The total length of haplotype blocks varied greatly from chromosome to chromosome. The total lengths of haplotype blocks in the A subgenome varied between 1,868 kb on A3 and 7,422 kb on A9. In the C subgenome, total length of haplotype blocks varied between 7,380 kb on C5 and 17,588 kb on C3 (Table 2). The haplotype blocks may result from selections against agronomically important traits in breeding programs. Rapeseed meal is a good source of protein for animal feed. However, glucosinolates are secondary metabolites in seeds with decomposition products that are toxic to animals. Low glucosinolate content has been a target trait for selection in rapeseed breeding programs since 1970’s [37]. One haplotype block on A2 extended for a physical distance of 636 kb (13,055–13,691 kb), which contained two genes (*BnaA02g20840D* and *BnaA02g20860D*) related to the biosynthesis of glucosinolates (Fig 5). *BnaA02g20840D* is homologous to *AT5G23010* (*MAM1*) in Arabidopsis and encodes a methylthioalkylmalate synthase that catalyzes the condensation reactions of the first two rounds of methionine chain elongation in the biosynthesis of methionine-derived glucosinolates [38]. *BnaA02g20860D* is homologous to *AT4G03060* (*AOP2*) in Arabidopsis and encodes a 2-oxoglutarate-dependent dioxygenase which is involved in glucosinolate biosynthesis [38]. Notably, the genetic diversity of SNPs within this haplotype block were significantly lower than that of the SNPs flanking the haplotype block (0.27 vs. 0.31, *P* = 0.001). The two SNPs located between *BnaA02g20840D* and *BnaA02g20860D* (Fig 5) had the lowest genetic diversity. These results suggested that the large haplotype block harboring these two genes perhaps experienced strong selection in breeding for low glucosinolate content oilseed rape [17].

### Relationship between geographic distribution and genome-wide haplotype blocks in *B*. *napus* germplasm

The 189 *B*. *napus* inbred lines in the association panel were collected from diverse origins over the world (S1 Table). To infer the ancestry relationships among the 189 inbred lines, we performed a Bayesian model-based population structure analysis with 2,562 unlinked SNPs that were selected from 10,343 SNPs using a sliding window strategy [26]. Two main groups were identified in the 189 inbred lines and designated as P1 and P2. Clear differentiation between these two groups was also verified by PCA (S2 Fig). The P1 group contained 136 lines, most of which originated from China and represented the semi-winter ecotype of oilseed rape. The P2 group contained 53 lines, most of which originated from Europe, Canada and Australia and mainly belonged to the spring ecotype (S1 Table, S2 Fig). The P1 was divided into two subgroups, G1 and G2, that contained 81 and 55 lines, respectively, and the P2 group was divided into two subgroups, named G3 and G4, which had 35 and 18 lines (S1 Table, S3 Fig). Because the sample sizes of subgroups were too small to obtain reliable results, the following analyses were only performed within and between the P1 and P2 groups.

Genetic diversity levels were measured as polymorphic informative content (PIC) values in the P1 and P2 groups. In the A subgenome, the PIC value of P1 was significantly higher than that of P2 (0.255 vs. 0.234, *P* < 0.001), whilst in the C subgenome the PIC value of P1 was significantly lower than that of P2 (0.214 vs. 0.275, *P* < 0.001) (S4 Table). We then analyzed the genome-wide haplotype patterns in each group. Across the whole genome 241 common haplotype blocks with total length of 91.2 Mb were identified. Among them 24 haplotype blocks with a total length of 5.3 Mb were group-preferential (Table 2). Furthermore, 206 and 180 group-specific haplotype blocks were identified for P1 and P2, respectively. These haplotype blocks were unevenly distributed across the *B*. *napus* genome (Fig 4B). The total lengths of the group-specific haplotype blocks in the C subgenome were 38.5 and 31.3 Mb for P1 and P2, respectively, which were about twice folds of those in the A subgenome (16.3 and 18.9 Mb for P1 and P2, respectively) (Table 2), suggesting that the C subgenome experienced stronger selection during breeding. Interestingly, the total length of P1-specific haplotype blocks in the A subgenome was shorter than that of P2-specific haplotype blocks, while the total length of P1-specific haplotype blocks in the C subgenome was longer than that of P2-specific haplotype blocks (Table 2).

### Dissection of oil content by GWAS

Extensive variation was observed for seed oil content in the panel. In 2009, the oil contents were from 32.7% to 47.8% with an average of 41.2%, and they ranged from 29.5% to 46.3% and 31.4% to 46.5%, with averages of 40.1% and 40.6% in 2010 and 2011, respectively (S4 Fig, S5 Table). Shapiro-Wilk normality test indicated that the oil content of seeds was not normally distributed (*P* value raged from 3.3×10^-4^ to 1.6×10^-2^) and the overall level skewed toward higher values (S4 Fig), which is consistent with the fact that many of the inbred lines are improved varieties. The broad-sense heritability (*H*^2^) for oil content was 87.7% (S5 Table), suggesting that it was stably inherited.

**Fig 6:**
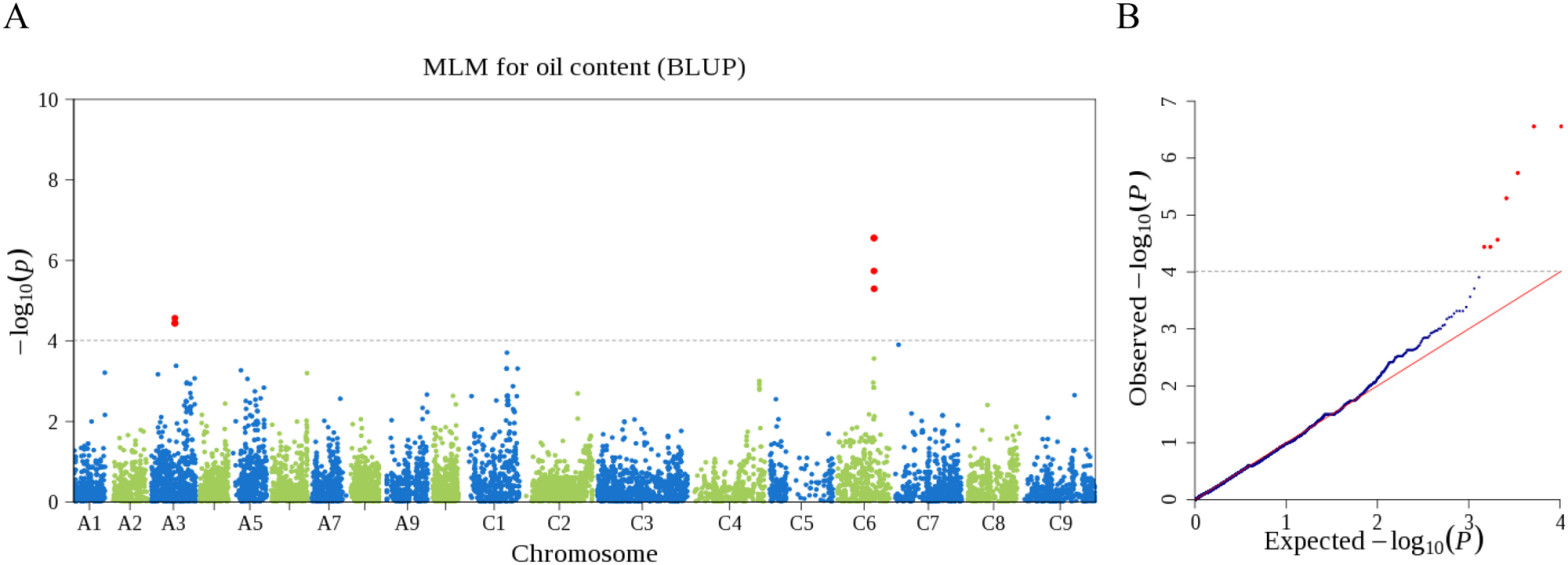
Manhattan and quantile-quantile plots of GWAS for seed oil content. The BLUP values of oil content across three years were used as phenotypes in GWAS. **(A)** Manhattan plot for oil content. The dashed horizontal line indicates the Bonferroni-adjusted significance threshold (*P* = 9.7×10^-5^). Red dots above the threshold indicate the significant SNPs for oil content. **(B)** Quantile-quantile plot for oil content.

GWAS of oil content was conducted for independent environment. Six, five and six SNPs were detected to be significantly associated with oil content in years 2009, 2010 and 2011 (*P* < 9.7×10^-5^, S6 Table), which represented two, three and three loci, respectively. Among them, two loci, *qOCA3* on A3 and *qOCC6* on C6, were repeatedly detected in all three environments (-log_10_*P* values were 4.05, 4.30 and 4.79 for *qOCA3*, and 4.86, 4.86 and 4.16 for *qOCC6* in 2009, 2010, 2011, respectively), suggesting that these loci were stably expressed in different environments and controlled oil accumulation in seeds. The other two loci, *qOCC1* and *qOCA6*, were specific to the environment of years 2010 and 2011, respectively. In addition, GWAS with the BLUP values of seed oil content across three environments also detected *qOCA3* and *qOCC6*, but missed the two environment-specific loci, *qOCC1* and *qOCA6* (Fig 6, S6 Table). Based on the stepwise regression analysis for all significant loci coupled with corresponding phenotype in each year, we observed that 17.0%, 21.5% and 23.6% of phenotypic variations were explained by all loci in 2009, 2010 and 2011, respectively (S6 Table).

The *qOCA3* locus had four haplotypes. Haplotype1 (H1) was the major and favorable haplotype with 168 (88.9%) inbred lines and had an average oil content of 40.92±1.88% (S7 Table), which is significantly higher than the average oil content of the other three haplotypes (38.00±2.85%, *P* = 1.4×10^-4^) (S7 Table). The *qOCC6* locus had two haplotypes, H1 (93.1%) being the major and favorable haplotype. Inbred lines conferring the H1 haplotype had an average oil content of 40.8±2.05%, which was significantly higher than that of H2 (37.34%±2.66%, *P* = 4.4×10^-4^).

## Discussion

*B*. *napus* is an allotetraploid species that originated from the natural hybridization of *B*. *rapa* and *B*. *oleracea*. Before this hybridization, the two progenitors had experienced whole genome triplication [20,39,40], which resulted in the existence of vast amounts of homologous and paralogous sequences in the *B*. *napus* genome. The existence of homologous and paralogous sequences makes it still difficult to discover SNPs even though the draft reference genome sequence of *B*. *napus* is available [20]. To avoid the disturbance of homologous and paralogous sequence in SNP discovery, the UNEAK pipeline only made use of networks containing two tags for SNP discovery [22]. Discarding networks containing multiple tags significantly simplified the process of SNP discovery, but it would definitely loss a large number of real SNPs. In addition, simply treating the reciprocal tags as two alleles from the same locus would unavoidably introduce false genotyping, because the reciprocal tags within a network are also probably to be inter-homolog polymorphisms (IHPs). In this study, we sequenced the association panel comprised of 189 inbred lines using the ddRAD technique and employed a modified UNEAK pipeline for SNP mining and genotyping. Compared to the original UNEAK pipeline [22], the modified UNEAK includes an additional step to discriminate allelic tags from paralogous and/or homeologous tags within a network by using relative heterozygosity in the association panel. With this step, allelic tags were discriminated not only from networks with two tags, but also from networks containing multiple tags (>2). In this study, 8,409 allelic tag pairs were mined from complex networks comprising of 3–10 tags, which was close to the number of SNPs identified from networks only containing two tags. These results suggest that the additional step is effective to discriminate allelic tags from paralogous and/or homologous tags. Thus the modified UNEAK pipeline is useful for SNP mining and genotyping in polyploidy plant species.

High-density SNP mapping provides an opportunity to characterize genome-wide LD patterns systematically in *B*. *napus*, which provides deep insights into breeding history in the past century and facilitates understanding the genetic determinants of complex traits in GWAS. Previously, a set of 451 SSR markers were used to evaluate LD level of the association panel, but the resolution of estimated LD level was low due to the limited number of markers [14]. In the present study, the genome-wide LD pattern of the same association panel was explored more clearly using 10,343 SNPs, which provided a valuable resource for association mapping. In the association panel of 189 *B*. *napus* inbred lines, the whole-genome LD decay distance was estimated to be 1,214 kb at the background level (*r*^2^ < 0.26), which was longer than the distances of LD decay estimated in previous studies using different *B*. *napus* representative germplasm resources [15–17]. In addition, LD in the C subgenome decayed much slower than in the A subgenome, which was congruent with the previous findings [15–17]. In this study, the C subgenome had more and longer haplotype blocks (>100 kb) than the A subgenome. These results implied that the stronger selection pressure had been applied in the C subgenome than the A subgenome since domestication in oilseed rape, which resulted in the obviously depressed genetic diversity on the C subgenome relative to the A subgenome.

The SNP markers clearly classified the association panel into two groups (P1 and P2) and four subgroups. The classification of the 189 inbred lines was basically consistent with that revealed using SSR markers [14]. Ten inbred lines were found to belong to different groups based on two set of markers. Of them, seven lines were shifted from P1 to P2, three lines from P2 to P1. This classification was based on much more and evenly distributed markers and was thought to be more reasonable. Inbred lines in P1 and P2 mainly represent semi-winter and spring type rapeseeds, respectively, which are consistent with their geographical distributions. In the whole genome, a large number of group-specific haplotype blocks were identified, suggesting that these genomic regions had been experienced distinct selections for adaptation to climates in different geographic regions. It was worth to note that the P1 group had more and longer haplotype blocks than the P2 group in the C subgenome, but it had less and shorter haplotype blocks than the P2 group in the A subgenome, suggesting that inbred lines in the P1 group had experienced a different breeding history from those in the P2 group. Inbred lines in the P1 group are mostly semi-winter type oilseed rape from China. The semi-winter type oilseed rape is a special ecotype adapted to the climates in the regions along the Yangtse River and is bred from extensive hybridization between the original oilseed rape introduced from Europe and Canada and local Chinese *B*. *rapa* cultivars [41] that has been cultivated as vegetables and oilcrop for more than 6,000 years in China. Therefore, the massive genetic introgression from *B*. *rapa* had largely increased the level of genetic diversity in the A subgenome and thus resulted in less and shorter haplotype blocks in P1 compared to P2. Additionally, the limited number of cultivars introduced from Europe or Canada in different periods were probably the founders of the C subgenome in these semi-winter cultivars. No exotic genome was further introgressed into the C subgenome of the semi-winter Chinese oilseed rape, which resulted in more and longer haplotype blocks in P1 than in P2 in the C subgenome. The understanding of the breeding history of oilseed rape in China would prompt us that it is necessary and urgent to broaden the level of genetic diversity in the C subgenome.

GWAS was performed on seed oil content using the high-density SNP markers. The two stably expressed loci for seed oil content, *qOCA3* and *qOCC6*, were also detected in previous studies using traditional linkage mapping [42, 43]. The favorable haplotypes, H1 (TAA) at *qOCA3* and H1 (GAAG) at *qOCC6*, have a frequency of 88.9% and 93.1%, respectively. Both favorable haplotypes had significantly higher oil content than the minor haplotypes. These results suggest that strong selection has been applied to these two favorable haplotypes. In the 200 kb region flanking *qOCA3*, a gene *BnaA03g32620D* encoding a lipid transport superfamily protein [44] was identified to be 54 kb from the peak SNP (snp01597). In the flanking region of *qOCC6*, a gene *BnaC06g22630D* encoding an acyltransferase and involving in diacylglycerol biosynthetic process [45] was found to be 185 kb from the peak SNP (snp12454). These results of GWAS demonstrated that the high-density SNPs developed in present study provided an effective tool to dissect the genetic architecture of quantitative traits in *B*. *napus*, although additional evidence is needed to support the identified loci and candidate genes. The SNPs associated with oil content would be useful for oil content improvement in oilseed rape breeding program.

## Supporting Information

S1 Fig Estimation of background LD level of the panel. The vertical dash red line indicates the 99^th^ percentile of *r*^2^ distribution for unlinked SNP pairs based on the genetic map with 4,995 anchor SNPs.
(TIF)

S2 Fig Population structure and principal component analysis (PCA). **(A)** Estimation of LnP(D) and Δ*k* in the whole panel. **(B)** The 189 diverse inbred lines were classified into two groups P1 and P2 by Structure analysis. **(C)** PCA of the entire panel, blue and red dots represent the inbred lines from P1 and P2, respectively, and the values in parenthesis represent the proportions of variances explained by the first two principal components.
(TIF)

S3 Fig Subgroups derived from P1 and P2 by Structure. Estimation of LnP(D) and Δ*k* for P1 **(A)** and P2 **(B)** in Structure. Subgroups were derived from P1 **(C)** and P2 **(D)**.
(TIF)

S4 Fig The distribution of seed oil contents in 189 inbred lines for each year and BLUP. (TIF)

S1 Table List of 189 inbred lines of *B*. *napus* and assignment for group and subgroup based on Structure analysis. (DOCX)

S2 Table Number of networks and allelic tags identified in the association panel using modified UNEAK pipeline. (DOCX)

S3 Table SNPs assigned to chromosomes by LD-based mapping and sequence alignment to the reference sequence. (DOCX)

S4 Table Genetic diversity and proportions of intra-chromosomal SNP pairs showing LD in the entire association panel and inferred groups. (DOCX)

S5 Table Descriptive statistics and broad-sense heritability for seed oil content. (DOCX)

S6 Table Summary of GWAS results for seed oil content. (DOCX)

S7 Table Haplotype analysis of *qOCA3* and *qOCC6* for seed oil content in *B*. *napus*. (DOCX)
