# Supplementary figures and images for: Evaluation of Linkage Disequilibrium Pattern and Association Study on Seed Oil Content in Brassica napus Using ddRAD Sequencing
Source: PLoS One. 2016 Jan 5;11(1):e0146383. doi: 10.1371/journal.pone.0146383 (PMC4701484; doi:10.1371/journal.pone.0146383)

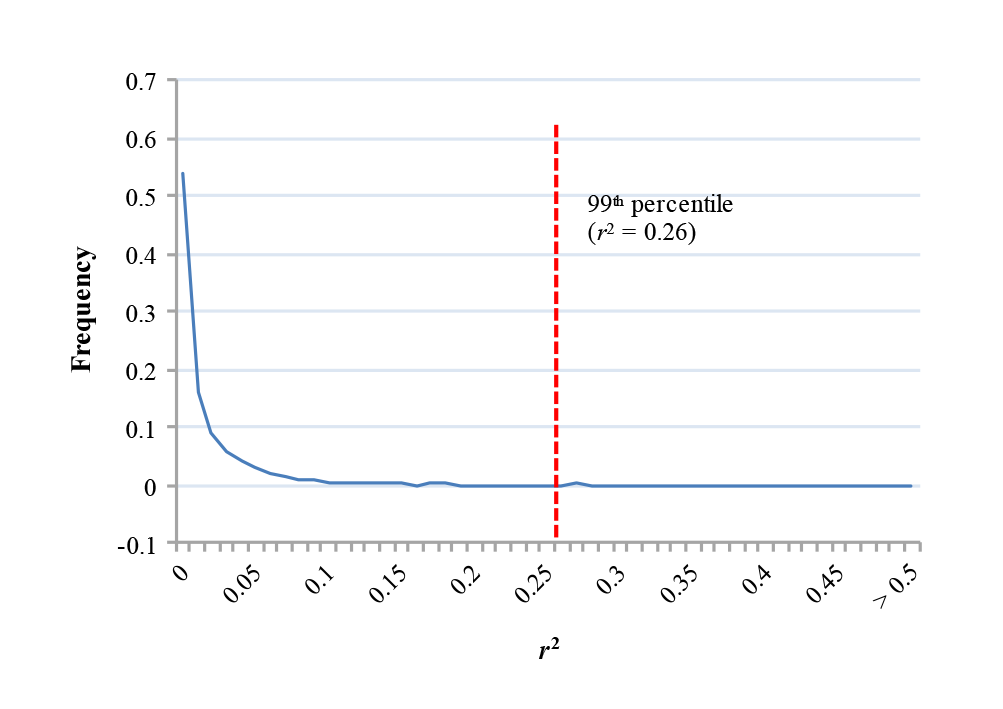

Supplement: S1 Fig — The vertical dash red line indicates the 99th percentile of r2 distribution for unlinked SNP pairs based on the genetic map with 4,995 anchor SNPs. (TIF) [file pone.0146383.s001.tif]

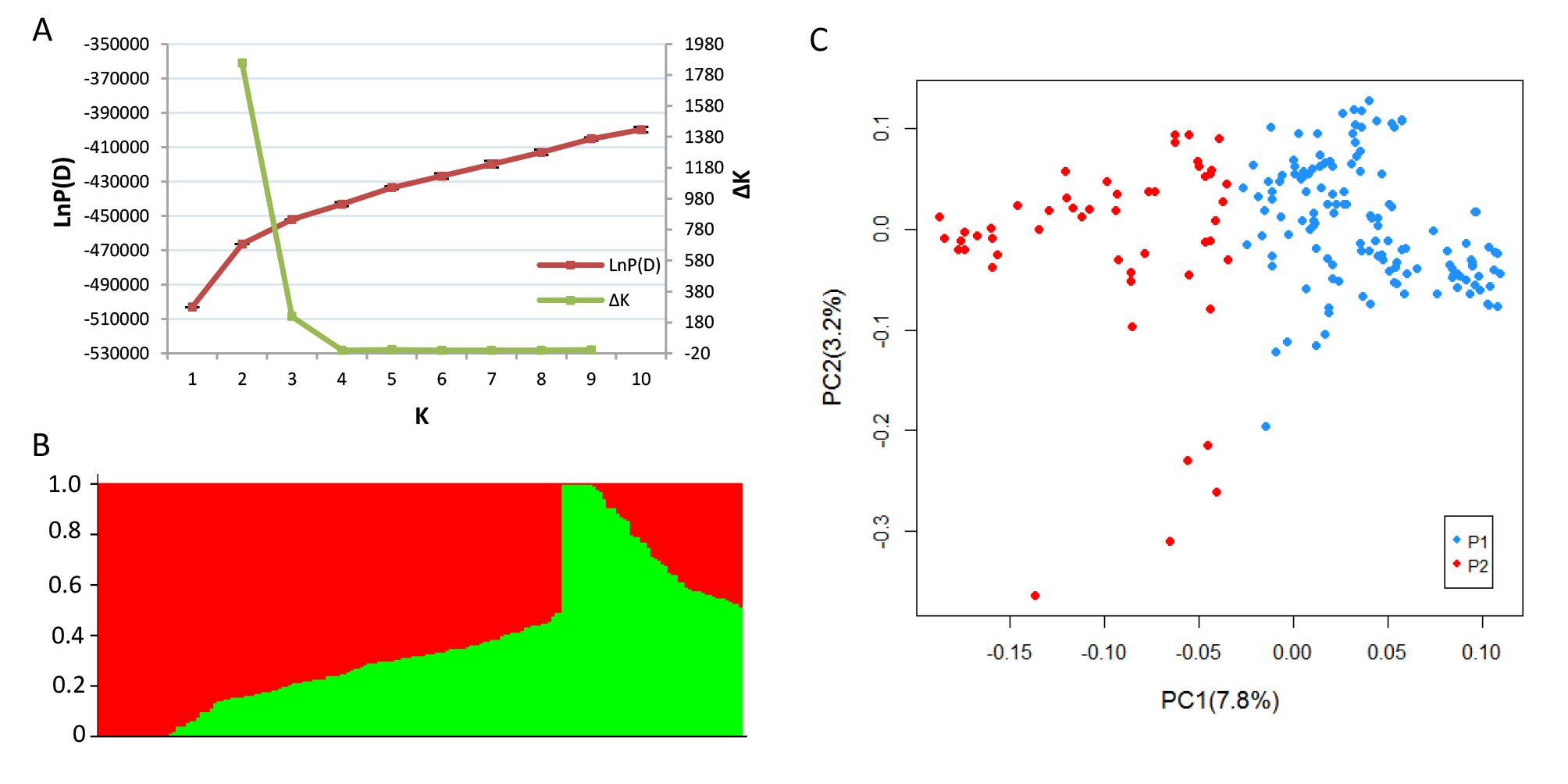

Supplement: S2 Fig — (A) Estimation of LnP(D) and Δk in the whole panel. (B) The 189 diverse inbred lines were classified into two groups P1 and P2 by Structure analysis. (C) PCA of the entire panel, blue and red dots represent the inbred lines from P1 and P2, respectively, and the values in parenthesis represent the proportions of variances explained by the first two principal components. (TIF) [file pone.0146383.s002.tif]

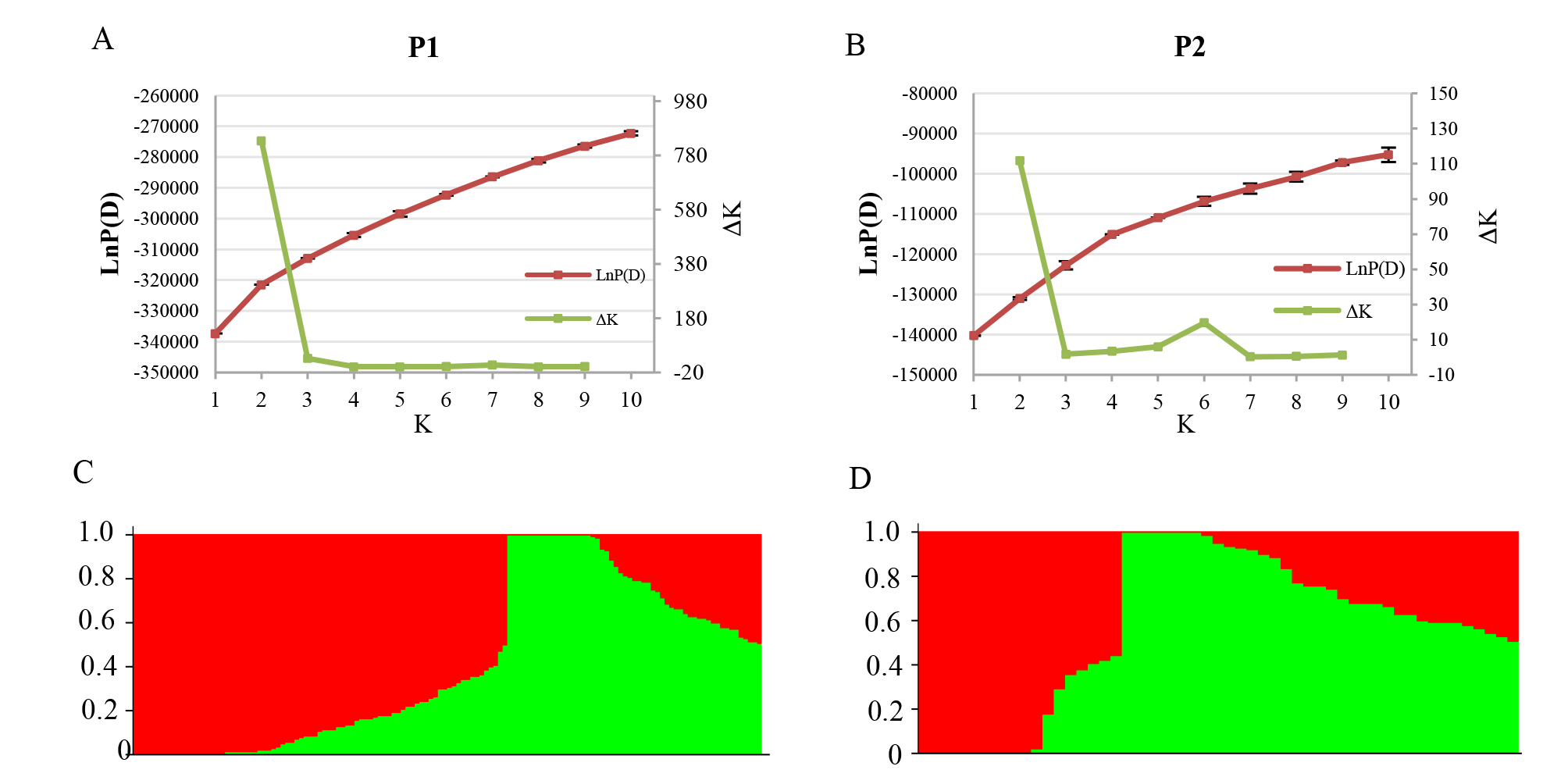

Supplement: S3 Fig — Estimation of LnP(D) and Δk for P1 (A) and P2 (B) in Structure. Subgroups were derived from P1 (C) and P2 (D). (TIF) [file pone.0146383.s003.tif]

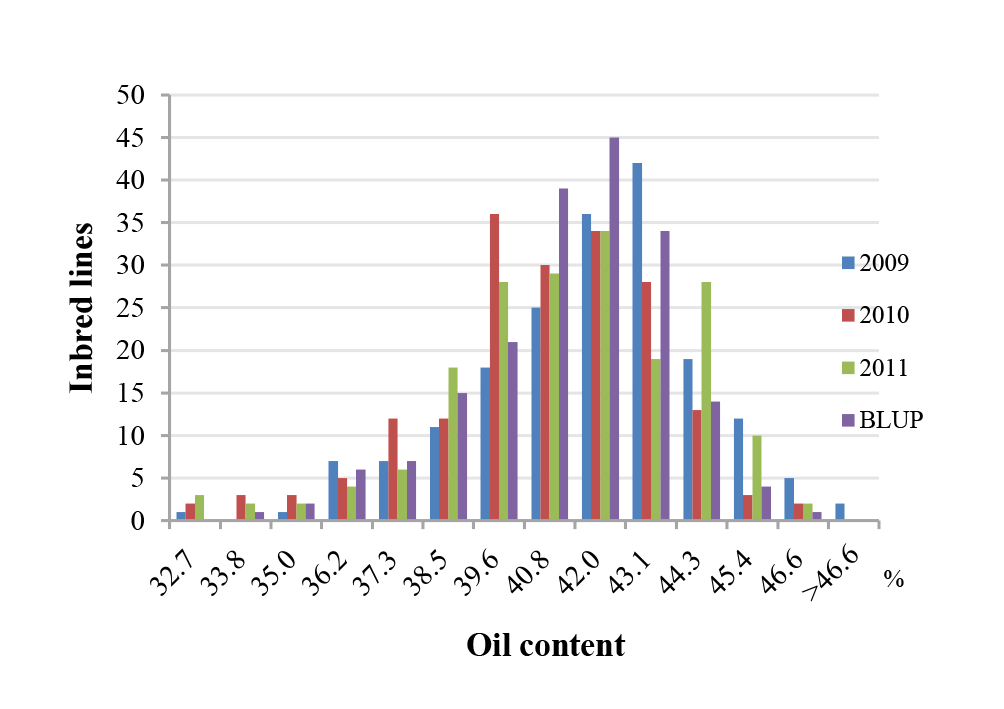

Supplement: S4 Fig — (TIF) [file pone.0146383.s004.tif]
