# Supplementary material for: Evaluation of Linkage Disequilibrium Pattern and Association Study on Seed Oil Content in Brassica napus Using ddRAD Sequencing
Source: PLoS One. 2016 Jan 5;11(1):e0146383. doi: 10.1371/journal.pone.0146383 (PMC4701484; doi:10.1371/journal.pone.0146383)
Supplement: S1 Table — (DOCX) [file pone.0146383.s005.docx]

| **S1 Table. List of 189 inbred lines of *B. napus* and assignment for group and subgroup based on Structure analysis.**   \| **Code** \| **Inbred line** \| **Geographical**  **region** \| **Ecotype** \| **Group assignment** \| **Subgroup assignment** \| \| --- \| --- \| --- \| --- \| --- \| --- \| \| g001 \| Zhongshuang2 \| Hubei (China) \| Semi-winter \| P1 \| G1 \| \| g002 \| Zhongza-H8002 \| Hubei (China) \| Spring \| P2 \| G3 \| \| g003 \| YD18(W) \| Hubei (China) \| Semi-winter \| P1 \| G1 \| \| g004 \| Zhongyou9636 \| Hubei (China) \| Semi-winter \| P1 \| G1 \| \| g005 \| Qingyou331 \| Qinghai (China) \| Semi-winter \| P1 \| G1 \| \| g006 \| 2000V57 \| Chongqing (China) \| Semi-winter \| P2 \| G3 \| \| g007 \| Impulse \| Australia \| Spring \| P2 \| G4 \| \| g008 \| Dunkeld \| Australia \| Spring \| P2 \| G3 \| \| g009 \| Rainbow \| Australia \| Spring \| P1 \| G1 \| \| g010 \| Monty \| Australia \| Spring \| P1 \| G1 \| \| g011 \| A1066 \| Hubei (China) \| Semi-winter \| P2 \| G3 \| \| g012 \| A2066 \| Hubei (China) \| Semi-winter \| P2 \| G3 \| \| g013 \| 25P34-1 \| Hubei (China) \| Semi-winter \| P1 \| G1 \| \| g014 \| Yunyou5 \| Yunnan (China) \| Semi-winter \| P1 \| G1 \| \| g015 \| Xiangyou15 \| Hunan (China) \| Semi-winter \| P1 \| G1 \| \| g016 \| Huashuang5 \| Hubei (China) \| Semi-winter \| P1 \| G1 \| \| g017 \| Zhongshuang9 \| Hubei (China) \| Semi-winter \| P1 \| G1 \| \| g018 \| H090 \| Yunnan (China) \| Semi-winter \| P1 \| G1 \| \| g019 \| Qinyou7 \| Shanxi (China) \| Spring \| P2 \| G3 \| \| g020 \| Ienvenu \| Canada \| Unknown \| P1 \| G1 \| \| g021 \| Norin24 \| Japan \| Semi-winter \| P2 \| G3 \| \| g022 \| EBONY \| Canada \| Spring \| P2 \| G3 \| \| g023 \| Surpass-400 \| Australia \| Spring \| P2 \| G3 \| \| g024 \| Quantum(oo) \| Canada \| Spring \| P2 \| G4 \| \| g025 \| Sprint(oo) \| Europe \| Spring \| P2 \| G3 \| \| g026 \| G1028 \| Europe \| Spring \| P2 \| G4 \| \| g027 \| SC-UG3 \| Canada \| Spring \| P2 \| G3 \| \| g028 \| Pinnade(TT) \| Australia \| Unknown \| P1 \| G1 \| \| g029 \| Drum(TT) \| Canada \| Spring \| P2 \| G3 \| \| g030 \| G1032 \| Hubei (China) \| Semi-winter \| P1 \| G2 \| \| g031 \| Wuxue2004 \| Hubei (China) \| Semi-winter \| P1 \| G2 \| \| g032 \| SC-UG6 \| Hubei (China) \| Spring \| P2 \| G4 \| \| g033 \| Bugle(TT) \| Canada \| Spring \| P2 \| G3 \| \| g034 \| Karod(TT) \| Canada \| Spring \| P2 \| G3 \| \| g035 \| G1037 \| Hubei (China) \| Semi-winter \| P1 \| G1 \| \| g036 \| G1038 \| Hubei (China) \| Semi-winter \| P1 \| G1 \| \| g037 \| Surpass-600(TT) \| Australia \| Spring \| P2 \| G3 \| \| g038 \| Roundup \| Canada \| Spring \| P2 \| G4 \| \| g039 \| Alto(oo) \| Australia \| Spring \| P1 \| G1 \| \| g040 \| 25CC3 \| Australia \| Spring \| P2 \| G4 \| \| g041 \| 25CC1 \| Australia \| Spring \| P2 \| G4 \| \| g042 \| G1044 \| Europe \| Spring \| P1 \| G2 \| \| g043 \| Huyou16(W) \| Shanghai (China) \| Semi-winter \| P2 \| G3 \| \| g044 \| Xiwang106 \| Hubei (China) \| Semi-winter \| P1 \| G1 \| \| g045 \| 24101 \| Hubei (China) \| Semi-winter \| P1 \| G1 \| \| g046 \| Fuyou2(W) \| Sichuan (China) \| Spring \| P1 \| G1 \| \| g047 \| Fan15 \| Hubei (China) \| Semi-winter \| P1 \| G1 \| \| g048 \| Deyou890 \| Sichuan (China) \| Semi-winter \| P1 \| G1 \| \| g049 \| 25yangsheng \| Hubei (China) \| Semi-winter \| P1 \| G1 \| \| g050 \| Chuan91 \| Sichuan (China) \| Semi-winter \| P1 \| G1 \| \| g051 \| DS \| Hubei (China) \| Semi-winter \| P1 \| G1 \| \| g052 \| Huyou14 \| Shanghai (China) \| Semi-winter \| P1 \| G1 \| \| g053 \| Huyou15 \| Shanghai (China) \| Semi-winter \| P1 \| G1 \| \| g054 \| Huyou16(M) \| Shanghai (China) \| Semi-winter \| P1 \| G1 \| \| g055 \| Huxiuqing \| Shanghai (China) \| Semi-winter \| P1 \| G1 \| \| g056 \| Shenyouqing \| Shanghai (China) \| Semi-winter \| P1 \| G2 \| \| g057 \| G1060 \| Hubei (China) \| Semi-winter \| P1 \| G1 \| \| g058 \| G1061 \| Hubei (China) \| Semi-winter \| P1 \| G1 \| \| g059 \| G1062 \| Hubei (China) \| Semi-winter \| P1 \| G1 \| \| g060 \| G1063 \| Hubei (China) \| Semi-winter \| P1 \| G1 \| \| g061 \| G1064 \| Hubei (China) \| Semi-winter \| P1 \| G1 \| \| g062 \| G1065 \| Hubei (China) \| Semi-winter \| P1 \| G1 \| \| g063 \| G1066 \| Hubei (China) \| Semi-winter \| P1 \| G1 \| \| g064 \| G1067 \| Hubei (China) \| Semi-winter \| P1 \| G1 \| \| g065 \| G1068 \| Hubei (China) \| Semi-winter \| P1 \| G1 \| \| g066 \| G1069 \| Hubei (China) \| Semi-winter \| P1 \| G1 \| \| g067 \| G1070 \| Hubei (China) \| Semi-winter \| P2 \| G4 \| \| g068 \| G1071 \| Hubei (China) \| Semi-winter \| P1 \| G1 \| \| g069 \| G1072 \| Hubei (China) \| Semi-winter \| P1 \| G1 \| \| g070 \| G1073 \| Hubei (China) \| Semi-winter \| P1 \| G1 \| \| g071 \| G1074 \| Hubei (China) \| Semi-winter \| P1 \| G1 \| \| g072 \| G1075 \| Hubei (China) \| Semi-winter \| P1 \| G1 \| \| g073 \| G1076 \| Hubei (China) \| Semi-winter \| P1 \| G1 \| \| g074 \| G1077 \| Hubei (China) \| Semi-winter \| P1 \| G1 \| \| g075 \| G1078 \| Hubei (China) \| Semi-winter \| P1 \| G1 \| \| g076 \| G1080 \| Hubei (China) \| Semi-winter \| P1 \| G1 \| \| g077 \| G1081 \| Hubei (China) \| Semi-winter \| P1 \| G1 \| \| g078 \| G1082 \| Hubei (China) \| Semi-winter \| P1 \| G1 \| \| g079 \| G1083 \| Hubei (China) \| Semi-winter \| P1 \| G1 \| \| g080 \| Huashuang4 \| Hubei (China) \| Semi-winter \| P1 \| G1 \| \| g081 \| G1085 \| Hubei (China) \| Semi-winter \| P2 \| G3 \| \| g082 \| 1728-1 \| Hubei (China) \| Semi-winter \| P1 \| G1 \| \| g083 \| G1087 \| Hubei (China) \| Semi-winter \| P2 \| G3 \| \| g084 \| 25Z0065-1(W) \| Hubei (China) \| Semi-winter \| P1 \| G1 \| \| g085 \| 24R14 \| Hubei (China) \| Semi-winter \| P1 \| G2 \| \| g086 \| 257052 \| Hubei (China) \| Semi-winter \| P2 \| G3 \| \| g087 \| 25F371-1 \| Hubei (China) \| Semi-winter \| P2 \| G3 \| \| g088 \| Rongyou4 \| Sichuan (China) \| Semi-winter \| P1 \| G1 \| \| g089 \| Mianyou12 \| Sichuan (China) \| Semi-winter \| P1 \| G1 \| \| g090 \| Zheshuang3 \| Zhejiang (China) \| Semi-winter \| P1 \| G2 \| \| g091 \| Chuanyou58 \| Sichuan (China) \| Semi-winter \| P1 \| G1 \| \| g092 \| 324 \| Hubei (China) \| Semi-winter \| P1 \| G2 \| \| g093 \| 4014 \| Hubei (China) \| Semi-winter \| P1 \| G2 \| \| g094 \| 84001 \| Hubei (China) \| Semi-winter \| P1 \| G1 \| \| g095 \| 84004 \| Hubei (China) \| Semi-winter \| P2 \| G3 \| \| g096 \| Aijiazao \| Hunan (China) \| Semi-winter \| P1 \| G2 \| \| g097 \| Baihua \| Hubei (China) \| Semi-winter \| P1 \| G2 \| \| g098 \| Bokwok.B \| Europe \| Unknown \| P2 \| G3 \| \| g099 \| Bolko \| Europe \| Winter \| P2 \| G3 \| \| g100 \| Chengdu186 \| Chengdu (China) \| Semi-winter \| P1 \| G2 \| \| g101 \| Cibrabra \| Europe \| Spring \| P2 \| G3 \| \| g102 \| DSV-SR-10 \| Europe \| Spring \| P2 \| G4 \| \| g103 \| Huyou9(W) \| Shanghai (China) \| Semi-winter \| P1 \| G2 \| \| g104 \| Jiayou3 \| Canada \| Semi-winter \| P1 \| G2 \| \| g105 \| Ningyou7 \| Jiangsu (China) \| Semi-winter \| P1 \| G2 \| \| g106 \| Nonglin22 \| Japan \| Semi-winter \| P1 \| G2 \| \| g107 \| Nonglin40 \| Japan \| Semi-winter \| P1 \| G2 \| \| g108 \| Tieganqing \| China \| Semi-winter \| P1 \| G2 \| \| g109 \| Xinghuahuang \| China \| Semi-winter \| P1 \| G2 \| \| g110 \| Yunyou-8 \| Yunnan (China) \| Semi-winter \| P1 \| G2 \| \| g111 \| 4312huashuang1 \| Hubei (China) \| Semi-winter \| P1 \| G1 \| \| g112 \| 91806huashuang3 \| Hubei (China) \| Semi-winter \| P1 \| G1 \| \| g113 \| Bvonowshi-DH \| Europe \| Spring \| P2 \| G4 \| \| g114 \| NingRS-1 \| Jiangsu (China) \| Semi-winter \| P1 \| G2 \| \| g115 \| YD18(M) \| Hubei (China) \| Semi-winter \| P1 \| G2 \| \| g116 \| Ganyou12 \| Jiangxi (China) \| Semi-winter \| P1 \| G2 \| \| g117 \| Gannaizi-1 \| Hubei (China) \| Semi-winter \| P1 \| G2 \| \| g118 \| Guizhouqianxuan \| Guizhou (China) \| Semi-winter \| P1 \| G2 \| \| g119 \| Gulliver \| Europe \| Spring \| P2 \| G4 \| \| g120 \| H-30 \| Europe \| Winter \| P2 \| G3 \| \| g121 \| H-47 \| Europe \| Semi-winter \| P1 \| G1 \| \| g122 \| H-5 \| Europe \| Semi-winter \| P1 \| G2 \| \| g123 \| CAo3Ho-4 \| Canada \| Spring \| P2 \| G3 \| \| g124 \| Cao221167 \| Canada \| Spring \| P2 \| G3 \| \| g125 \| Zayuan1 \| Hubei (China) \| Semi-winter \| P1 \| G1 \| \| g126 \| Kangnongda \| Hubei (China) \| Semi-winter \| P1 \| G1 \| \| g127 \| 018A1 \| Australia \| Semi-winter \| P1 \| G1 \| \| g128 \| P315-1 \| Hubei (China) \| Semi-winter \| P2 \| G3 \| \| g129 \| G1135 \| Hubei (China) \| Semi-winter \| P1 \| G2 \| \| g130 \| Jia99H99-15NR \| Canada \| Spring \| P2 \| G4 \| \| g131 \| G1140 \| Hubei (China) \| Semi-winter \| P1 \| G1 \| \| g132 \| G1141 \| Hubei (China) \| Semi-winter \| P1 \| G2 \| \| g133 \| Jia99/98 \| Canada \| Spring \| P2 \| G4 \| \| g134 \| GanbaiF7 \| Hubei (China) \| Semi-winter \| P1 \| G1 \| \| g135 \| GanganF7 \| Hubei (China) \| Semi-winter \| P1 \| G1 \| \| g136 \| G1145 \| Hubei (China) \| Semi-winter \| P1 \| G1 \| \| g137 \| 25Z0065-1(M) \| Hubei (China) \| Semi-winter \| P1 \| G1 \| \| g138 \| No.2127-17 \| China \| Winter \| P2 \| G3 \| \| g139 \| ZY821 \| Hubei (China) \| Semi-winter \| P1 \| G2 \| \| g140 \| s1 \| Hubei (China) \| Semi-winter \| P1 \| G2 \| \| g141 \| s2 \| Hubei (China) \| Semi-winter \| P2 \| G3 \| \| g142 \| m201 \| Hubei (China) \| Semi-winter \| P1 \| G1 \| \| g143 \| m202 \| Hubei (China) \| Semi-winter \| P1 \| G1 \| \| g144 \| Bakworb \| Europe \| Spring \| P2 \| G3 \| \| g145 \| Bronowski \| Europe \| Spring \| P2 \| G4 \| \| g146 \| G1155 \| Hubei (China) \| Semi-winter \| P1 \| G2 \| \| g147 \| Chuanyou11 \| Sichuan (China) \| Semi-winter \| P1 \| G2 \| \| g148 \| Dac-chosen \| Europe \| Semi-winter \| P1 \| G2 \| \| g149 \| Rexi \| Europe \| Semi-winter \| P1 \| G1 \| \| g150 \| Fu2 \| China \| Semi-winter \| P1 \| G2 \| \| g151 \| Ganyou2 \| Jiangxi (China) \| Semi-winter \| P1 \| G2 \| \| g152 \| Ganyou3 \| Jiangxi (China) \| Semi-winter \| P1 \| G2 \| \| g153 \| Ganyou14 \| Jiangxi (China) \| Semi-winter \| P1 \| G1 \| \| g154 \| Guizhouqianxuan6 \| Guizhou (China) \| Semi-winter \| P1 \| G2 \| \| g155 \| Huayou11 \| Hubei (China) \| Semi-winter \| P1 \| G2 \| \| g156 \| Huayou14 \| Hubei (China) \| Semi-winter \| P1 \| G2 \| \| g157 \| Huayou4 \| Hubei (China) \| Semi-winter \| P1 \| G2 \| \| g158 \| Huayou5 \| Hubei (China) \| Semi-winter \| P1 \| G2 \| \| g159 \| Huayou6 \| Hubei (China) \| Semi-winter \| P1 \| G2 \| \| g160 \| Huayou10 \| Hubei (China) \| Semi-winter \| P1 \| G2 \| \| g161 \| Huyou9(M) \| Shanghai (China) \| Semi-winter \| P1 \| G2 \| \| g162 \| Jet-Ne21 \| Europe \| Spring \| P2 \| G3 \| \| g163 \| Naleo \| Europe \| Spring \| P2 \| G4 \| \| g164 \| Nilla \| Europe \| Spring \| P2 \| G4 \| \| g165 \| Primo \| Europe \| Spring \| P2 \| G4 \| \| g166 \| Puyou3 \| China \| Semi-winter \| P1 \| G2 \| \| g167 \| G1178 \| Japan \| Semi-winter \| P1 \| G2 \| \| g168 \| Wesvlay \| Europe \| Spring \| P2 \| G3 \| \| g169 \| Willi \| Europe \| Spring \| P2 \| G4 \| \| g170 \| Wuo \| Europe \| Semi-winter \| P1 \| G2 \| \| g171 \| Xiangnongyou2 \| Hunan (China) \| Semi-winter \| P1 \| G2 \| \| g172 \| Xiangnongyou3 \| Hunan (China) \| Semi-winter \| P1 \| G2 \| \| g173 \| Youguangye \| Hubei (China) \| Semi-winter \| P1 \| G2 \| \| g174 \| Ningyou7-DH3 \| Jiangsu (China) \| Semi-winter \| P1 \| G2 \| \| g175 \| Shan115-2-1 \| China \| Semi-winter \| P1 \| G1 \| \| g176 \| Zheyou7 \| Zhejiang (China) \| Semi-winter \| P1 \| G2 \| \| g177 \| Qingyou2 \| Qinghai (China) \| Semi-winter \| P1 \| G2 \| \| g178 \| Fuyou2(M) \| China \| Semi-winter \| P1 \| G2 \| \| g179 \| YP1-DH \| China \| Semi-winter \| P1 \| G2 \| \| g180 \| SV-pyriter \| Canada \| Semi-winter \| P1 \| G2 \| \| g181 \| Mricultureilctria \| Europe \| Spring \| P2 \| G3 \| \| g182 \| 308-2 \| China \| Semi-winter \| P1 \| G1 \| \| g183 \| 350 \| China \| Semi-winter \| P1 \| G1 \| \| g184 \| 352 \| China \| Semi-winter \| P1 \| G1 \| \| g185 \| 629-4 \| China \| Semi-winter \| P1 \| G1 \| \| g186 \| 529-1 \| China \| Semi-winter \| P2 \| G3 \| \| g187 \| 264-1 \| China \| Semi-winter \| P1 \| G1 \| \| g188 \| 473-1 \| China \| Semi-winter \| P1 \| G1 \| \| g189 \| 473-2 \| China \| Semi-winter \| P1 \| G1 \| |  |  |  |  |  |
| --- | --- | --- | --- | --- | --- | --- | --- | --- | --- | --- | --- | --- | --- | --- | --- | --- | --- | --- | --- | --- | --- | --- | --- | --- | --- | --- | --- | --- | --- | --- | --- | --- | --- | --- | --- | --- | --- | --- | --- | --- | --- | --- | --- | --- | --- | --- | --- | --- | --- | --- | --- | --- | --- | --- | --- | --- | --- | --- | --- | --- | --- | --- | --- | --- | --- | --- | --- | --- | --- | --- | --- | --- | --- | --- | --- | --- | --- | --- | --- | --- | --- | --- | --- | --- | --- | --- | --- | --- | --- | --- | --- | --- | --- | --- | --- | --- | --- | --- | --- | --- | --- | --- | --- | --- | --- | --- | --- | --- | --- | --- | --- | --- | --- | --- | --- | --- | --- | --- | --- | --- | --- | --- | --- | --- | --- | --- | --- | --- | --- | --- | --- | --- | --- | --- | --- | --- | --- | --- | --- | --- | --- | --- | --- | --- | --- | --- | --- | --- | --- | --- | --- | --- | --- | --- | --- | --- | --- | --- | --- | --- | --- | --- | --- | --- | --- | --- | --- | --- | --- | --- | --- | --- | --- | --- | --- | --- | --- | --- | --- | --- | --- | --- | --- | --- | --- | --- | --- | --- | --- | --- | --- | --- | --- | --- | --- | --- | --- | --- | --- | --- | --- | --- | --- | --- | --- | --- | --- | --- | --- | --- | --- | --- | --- | --- | --- | --- | --- | --- | --- | --- | --- | --- | --- | --- | --- | --- | --- | --- | --- | --- | --- | --- | --- | --- | --- | --- | --- | --- | --- | --- | --- | --- | --- | --- | --- | --- | --- | --- | --- | --- | --- | --- | --- | --- | --- | --- | --- | --- | --- | --- | --- | --- | --- | --- | --- | --- | --- | --- | --- | --- | --- | --- | --- | --- | --- | --- | --- | --- | --- | --- | --- | --- | --- | --- | --- | --- | --- | --- | --- | --- | --- | --- | --- | --- | --- | --- | --- | --- | --- | --- | --- | --- | --- | --- | --- | --- | --- | --- | --- | --- | --- | --- | --- | --- | --- | --- | --- | --- | --- | --- | --- | --- | --- | --- | --- | --- | --- | --- | --- | --- | --- | --- | --- | --- | --- | --- | --- | --- | --- | --- | --- | --- | --- | --- | --- | --- | --- | --- | --- | --- | --- | --- | --- | --- | --- | --- | --- | --- | --- | --- | --- | --- | --- | --- | --- | --- | --- | --- | --- | --- | --- | --- | --- | --- | --- | --- | --- | --- | --- | --- | --- | --- | --- | --- | --- | --- | --- | --- | --- | --- | --- | --- | --- | --- | --- | --- | --- | --- | --- | --- | --- | --- | --- | --- | --- | --- | --- | --- | --- | --- | --- | --- | --- | --- | --- | --- | --- | --- | --- | --- | --- | --- | --- | --- | --- | --- | --- | --- | --- | --- | --- | --- | --- | --- | --- | --- | --- | --- | --- | --- | --- | --- | --- | --- | --- | --- | --- | --- | --- | --- | --- | --- | --- | --- | --- | --- | --- | --- | --- | --- | --- | --- | --- | --- | --- | --- | --- | --- | --- | --- | --- | --- | --- | --- | --- | --- | --- | --- | --- | --- | --- | --- | --- | --- | --- | --- | --- | --- | --- | --- | --- | --- | --- | --- | --- | --- | --- | --- | --- | --- | --- | --- | --- | --- | --- | --- | --- | --- | --- | --- | --- | --- | --- | --- | --- | --- | --- | --- | --- | --- | --- | --- | --- | --- | --- | --- | --- | --- | --- | --- | --- | --- | --- | --- | --- | --- | --- | --- | --- | --- | --- | --- | --- | --- | --- | --- | --- | --- | --- | --- | --- | --- | --- | --- | --- | --- | --- | --- | --- | --- | --- | --- | --- | --- | --- | --- | --- | --- | --- | --- | --- | --- | --- | --- | --- | --- | --- | --- | --- | --- | --- | --- | --- | --- | --- | --- | --- | --- | --- | --- | --- | --- | --- | --- | --- | --- | --- | --- | --- | --- | --- | --- | --- | --- | --- | --- | --- | --- | --- | --- | --- | --- | --- | --- | --- | --- | --- | --- | --- | --- | --- | --- | --- | --- | --- | --- | --- | --- | --- | --- | --- | --- | --- | --- | --- | --- | --- | --- | --- | --- | --- | --- | --- | --- | --- | --- | --- | --- | --- | --- | --- | --- | --- | --- | --- | --- | --- | --- | --- | --- | --- | --- | --- | --- | --- | --- | --- | --- | --- | --- | --- | --- | --- | --- | --- | --- | --- | --- | --- | --- | --- | --- | --- | --- | --- | --- | --- | --- | --- | --- | --- | --- | --- | --- | --- | --- | --- | --- | --- | --- | --- | --- | --- | --- | --- | --- | --- | --- | --- | --- | --- | --- | --- | --- | --- | --- | --- | --- | --- | --- | --- | --- | --- | --- | --- | --- | --- | --- | --- | --- | --- | --- | --- | --- | --- | --- | --- | --- | --- | --- | --- | --- | --- | --- | --- | --- | --- | --- | --- | --- | --- | --- | --- | --- | --- | --- | --- | --- | --- | --- | --- | --- | --- | --- | --- | --- | --- | --- | --- | --- | --- | --- | --- | --- | --- | --- | --- | --- | --- | --- | --- | --- | --- | --- | --- | --- | --- | --- | --- | --- | --- | --- | --- | --- | --- | --- | --- | --- | --- | --- | --- | --- | --- | --- | --- | --- | --- | --- | --- | --- | --- | --- | --- | --- | --- | --- | --- | --- | --- | --- | --- | --- | --- | --- | --- | --- | --- | --- | --- | --- | --- | --- | --- | --- | --- | --- | --- | --- | --- | --- | --- | --- | --- | --- | --- | --- | --- | --- | --- | --- | --- | --- | --- | --- | --- | --- | --- | --- | --- | --- | --- | --- | --- | --- | --- | --- | --- | --- | --- | --- | --- | --- | --- | --- | --- | --- | --- | --- | --- | --- | --- | --- | --- | --- | --- | --- | --- | --- | --- | --- | --- | --- | --- | --- | --- | --- | --- | --- | --- | --- | --- | --- | --- | --- | --- | --- | --- | --- | --- | --- | --- | --- | --- | --- | --- | --- | --- | --- | --- | --- | --- | --- | --- | --- | --- | --- | --- | --- | --- | --- | --- | --- | --- | --- | --- | --- | --- | --- | --- | --- | --- | --- | --- | --- | --- | --- | --- | --- | --- | --- | --- | --- | --- | --- | --- | --- | --- | --- | --- | --- | --- | --- | --- | --- | --- | --- | --- | --- | --- | --- | --- | --- | --- | --- | --- | --- | --- | --- | --- | --- | --- | --- | --- | --- | --- | --- | --- | --- | --- | --- | --- | --- | --- | --- | --- | --- | --- | --- | --- | --- | --- | --- | --- | --- | --- | --- | --- | --- | --- | --- | --- | --- | --- | --- | --- | --- | --- | --- | --- | --- | --- | --- | --- | --- | --- | --- | --- | --- | --- | --- | --- | --- | --- | --- | --- | --- | --- | --- | --- | --- | --- | --- | --- | --- | --- | --- | --- | --- | --- | --- | --- | --- | --- | --- | --- | --- | --- | --- | --- | --- | --- | --- | --- | --- | --- | --- | --- | --- | --- | --- | --- | --- | --- | --- | --- | --- | --- | --- | --- | --- | --- | --- | --- | --- | --- | --- | --- | --- | --- | --- | --- | --- | --- | --- | --- | --- | --- | --- | --- | --- | --- | --- | --- | --- | --- | --- | --- | --- | --- | --- | --- | --- | --- | --- | --- | --- | --- | --- | --- | --- | --- | --- | --- | --- | --- | --- | --- | --- | --- | --- | --- | --- | --- | --- | --- | --- | --- | --- | --- | --- | --- | --- | --- | --- | --- |
|  |  |  |  |  |  |
