# Supplementary material for: Evaluation of Linkage Disequilibrium Pattern and Association Study on Seed Oil Content in Brassica napus Using ddRAD Sequencing
Source: PLoS One. 2016 Jan 5;11(1):e0146383. doi: 10.1371/journal.pone.0146383 (PMC4701484; doi:10.1371/journal.pone.0146383)
Supplement: S2 Table — (DOCX) [file pone.0146383.s006.docx]

**S2 Table. Number of networks and allelic tags identified in the association panel using modified UNEAK pipeline.**

| Tags/  Network^a^ | No. networks^b^ | No. allelic tag pairs^c^ |
| --- | --- | --- |
| 1 | 583,375 | 0 |
| 2 | 90,235 | 10,918 |
| 3 | 26,002 | 4,140 |
| 4 | 11,023 | 1,945 |
| 5 | 5,405 | 929 |
| 6 | 3,321 | 547 |
| 7 | 2,232 | 312 |
| 8 | 1,765 | 229 |
| 9 | 1,318 | 168 |
| 10 | 1,087 | 139 |
| >10 | 7,484 |  |
| Total | 733,247 | 19,327 |

^a^ The number of unique tags per network identified by modified UNEAK pipeline.

^b^ The number of networks for each network type in the association panel.

^c^ The number of allelic tag pairs for each network type in the association panel.
