# Supplementary material for: Evaluation of Linkage Disequilibrium Pattern and Association Study on Seed Oil Content in Brassica napus Using ddRAD Sequencing
Source: PLoS One. 2016 Jan 5;11(1):e0146383. doi: 10.1371/journal.pone.0146383 (PMC4701484; doi:10.1371/journal.pone.0146383)
Supplement: S3 Table — (DOCX) [file pone.0146383.s007.docx]

**S3 Table. SNPs assigned to chromosomes by LD-based mapping and sequence alignment to the reference sequence.**

| **Chromosome** | **BnaNZDH^a^** | **LD mapped (%)^b^** | **aligned^c^** |
| --- | --- | --- | --- |
| A1 | 185 | 597(75.7) | 382 |
| A2 | 110 | 472(80.7) | 363 |
| A3 | 281 | 1,053(71.8) | 704 |
| A4 | 144 | 559(80.0) | 415 |
| A5 | 126 | 635(68.2) | 405 |
| A6 | 322 | 845(81.2) | 627 |
| A7 | 170 | 570(78.8) | 412 |
| A8 | 120 | 638(73.8) | 446 |
| A9 | 290 | 860(69.3) | 468 |
| A10 | 201 | 654(78.3) | 486 |
| C1 | 126 | 836(80.0) | 628 |
| C2 | 609 | 1,501(83.2) | 1,095 |
| C3 | 668 | 1,850(81.1) | 1,351 |
| C4 | 156 | 843(76.9) | 608 |
| C5 | 91 | 332(73.8) | 230 |
| C6 | 372 | 968(79.2) | 687 |
| C7 | 329 | 983(83.3) | 747 |
| C8 | 442 | 886(87.5) | 708 |
| C9 | 253 | 839(75.4) | 564 |
| A subgenome | 1,949 | 6,883(75.3) | 4,708 |
| C subgenome | 3,046 | 9,038(80.8) | 6,618 |
| Genome | 4,995 | 15,921(78.4) | 11,326 |

^a^ Number of SNPs in the original BnaNZDH genetic linkage map.

^b^ The number of SNPs assigned to each chromosome through LD-based mapping.

^c^ The number of SNPs aligned to a unique position of the draft reference genome sequence of *B. napus*.
