# Supplementary material for: Evaluation of Linkage Disequilibrium Pattern and Association Study on Seed Oil Content in Brassica napus Using ddRAD Sequencing
Source: PLoS One. 2016 Jan 5;11(1):e0146383. doi: 10.1371/journal.pone.0146383 (PMC4701484; doi:10.1371/journal.pone.0146383)
Supplement: S4 Table — (DOCX) [file pone.0146383.s008.docx]

**S4 Table. Genetic diversities and proportions of intra-chromosomal SNP pairs showing LD in the entire association panel and inferred groups.**

|  | **Panel** | | **P1** | | **P2** | |
| --- | --- | --- | --- | --- | --- | --- |
|  | **PIC** | **LD (%)^a^** | **PIC** | **LD (%)^a^** | **PIC** | **LD (%)^a^** |
| A subgenome | 0.269 | 7.41 | 0.255 | 7.53 | 0.234 | 8.34 |
| C subgenome | 0.251 | 14.64 | 0.214 | 13.90 | 0.275 | 15.14 |
| Genome | 0.259 | 13.73 | 0.232 | 11.82 | 0.257 | 14.67 |

^a^ The background LD level was set to *r*^2^ > 0.26 as described in Results.
