# Supplementary material for: Evaluation of Linkage Disequilibrium Pattern and Association Study on Seed Oil Content in Brassica napus Using ddRAD Sequencing
Source: PLoS One. 2016 Jan 5;11(1):e0146383. doi: 10.1371/journal.pone.0146383 (PMC4701484; doi:10.1371/journal.pone.0146383)
Supplement: S5 Table — (DOCX) [file pone.0146383.s009.docx]

**S5 Table. Descriptive statistics and broad-sense heritability for seed oil content.**

| **Year** | **Oil content (%)^a^** | **Range (%)** | ***P*_s_^b^** | **G^c^** | **E** | **G×E** | ***H*^2^ (%)^d^** |
| --- | --- | --- | --- | --- | --- | --- | --- |
| 2009 | 41.2±0.2 | 32.7-47.8 | 1.6×10^-2^ | ** | ** | ** | 87.7 |
| 2010 | 40.1±0.2 | 29.5-46.3 | 4.2×10^-4^ |  |  |  |  |
| 2011 | 40.6±0.2 | 31.4-46.5 | 3.3×10^-4^ |  |  |  |  |
| BLUP | 40.6±0.2 | 33.8-46.2 | 6.2×10^-4^ |  |  |  |  |

^a^ Mean±SD value for oil content.

^b^ Stands for *P* value of the Shapiro-Wilk test.

^c^ Significant at P < 0.01 (**) for the effect of genotype (G), environment (E) and genotype by environment interaction (G × E) on phenotypic variance estimated by ANOVA.

^d^ Family mean-based broad-sense heritability for oil contents of three years.
