# Supplementary material for: Evaluation of Linkage Disequilibrium Pattern and Association Study on Seed Oil Content in Brassica napus Using ddRAD Sequencing
Source: PLoS One. 2016 Jan 5;11(1):e0146383. doi: 10.1371/journal.pone.0146383 (PMC4701484; doi:10.1371/journal.pone.0146383)
Supplement: S6 Table — (DOCX) [file pone.0146383.s010.docx]

**S6 Table. Summary of GWAS results for seed oil content.**

| **Year** | **QTL** | **Chr.** | **SNP** | **Physical position^a^** | **Alleles** | **-log_10_*P*** | ***R*^2^ (%)^b^** |
| --- | --- | --- | --- | --- | --- | --- | --- |
| 2009 | *qOCA3* | A3 | snp01579 | 15,788,370 | A/T | 4.05 | 17.0 |
|  |  | A3 | snp01580 | 15,921,885 | G/A | 4.05 |  |
|  | *qOCC6* | C6 | snp12342 | 23,945,934 | A/G | 4.74 |  |
|  |  | C6 | snp12457 | 24,444,026 | A/G | 4.86 |  |
|  |  | C6 | snp12458 | 24,447,346 | G/A | 4.48 |  |
|  |  | C6 | snp12454 | 24,469,576 | G/A | 4.86 |  |
| 2010 | *qOCA3* | A3 | snp01597 | 15,817,158 | G/A | 4.30 | 21.5 |
|  | *qOCC1* | C1 | snp07436 | 29,083,332 | A/C | 4.04 |  |
|  |  | C1 | snp07435 | 29,243,429 | A/G | 4.04 |  |
|  | *qOCC6* | C6 | snp12457 | 24,444,026 | A/G | 4.86 |  |
|  |  | C6 | snp12454 | 24,469,576 | G/A | 4.86 |  |
| 2011 | *qOCA3* | A3 | snp01579 | 15,788,370 | A/T | 4.20 | 23.6 |
|  |  | A3 | snp01597 | 15,817,158 | G/A | 4.79 |  |
|  |  | A3 | snp01580 | 15,921,885 | G/A | 4.20 |  |
|  |  | A3 | snp01615 | 16,629,108 | T/G | 4.42 |  |
|  | *qOCA6* | A6 | snp04138 | 24,080,659 | G/A | 4.86 |  |
|  | *qOCC6* | C6 | snp12347 | 24,474,368 | G/A | 4.16 |  |
| BLUP | *qOCA3* | A3 | snp01579 | 15,788,370 | A/T | 4.44 | 27.8 |
|  |  | A3 | snp01597 | 15,817,158 | G/A | 4.57 |  |
|  |  | A3 | snp01580 | 15,921,885 | G/A | 4.44 |  |
|  | *qOCC6* | C6 | snp12457 | 24,444,026 | A/G | 6.56 |  |
|  |  | C6 | snp12458 | 24,447,346 | G/A | 5.74 |  |
|  |  | C6 | snp12454 | 24,469,576 | G/A | 6.56 |  |
|  |  | C6 | snp12455 | 24,533,014 | C/G | 5.30 |  |

^a^ Physical position of the SNP based on the genome sequence of Darmor-*bzh* v4.1.

^b^ Percentage of phenotypic variation explained by all significant loci using stepwise regression analysis.
