# Supplementary material for: Evaluation of Linkage Disequilibrium Pattern and Association Study on Seed Oil Content in Brassica napus Using ddRAD Sequencing
Source: PLoS One. 2016 Jan 5;11(1):e0146383. doi: 10.1371/journal.pone.0146383 (PMC4701484; doi:10.1371/journal.pone.0146383)
Supplement: S7 Table — (DOCX) [file pone.0146383.s011.docx]

**S7 Table. Haplotype analysis of *qOCA3* and *qOCC6* for seed oil content in *B. napus*.**

| **QTL** | **Haplotype** | **SNPs^a^** | **No. lines** | **Oil content (%)** | ***P* value** |
| --- | --- | --- | --- | --- | --- |
| *qOCA3* | H1 | TAA | 168 | 40.92±1.88 | 1.4×10^-4^ |
|  | H2/H3/H4 | AAG/ TGA/ AGG | 21 | 38.00±2.85 |  |
| *qOCC6* | H1 | GAAG | 176 | 40.80±2.05 | 4.4×10^-4^ |
|  | H2 | AGGC | 11 | 37.34±2.66 |  |

^a^ The haplotypes containing all significantly associated SNPs in each locus based on the GWAS using phenotypes of BLUP. The SNPs in each haplotype were sorted by the physical positions. The haplotype, which contained at least 2 inbred lines, was listed.
